# Metal Oxide Nanoparticles Synthesized in Ionic Liquids: Characterization and Photodegradation of Methyl Orange

**DOI:** 10.1021/acsomega.4c07627

**Published:** 2025-03-06

**Authors:** Mohamedalameen H. A. Hussain, Gulin Selda Pozan Soylu

**Affiliations:** Faculty of Engineering, Chemical Engineering Department, Istanbul University-Cerrahpaşa, Avcilar, 34320 Istanbul, Turkey

## Abstract

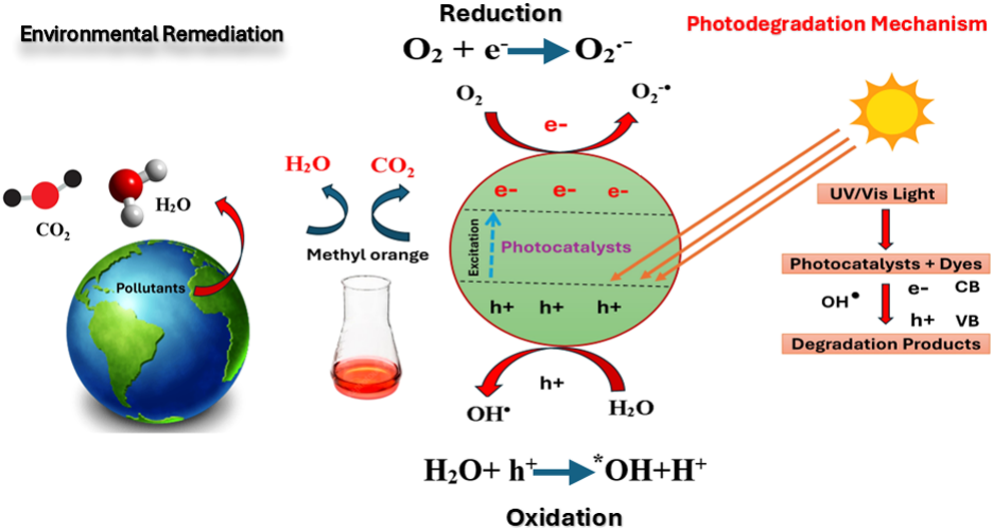

Dye residues from the textile industry significantly contribute to water pollution, necessitating effective wastewater treatment methods. This study reports the successful synthesis of zinc oxide (ZnO) nanoparticles using various ionic liquids (ILs), [BMIM]-BF_4_, [BMIM]-PF_6_, and [BMIM]-Cl, as mediators. The synthesized nanomaterials were characterized using various techniques, including X-ray diffraction (XRD), Fourier transform infrared (FTIR) spectroscopy, scanning electron microscopy (SEM), and photoluminescence (PL) spectroscopy. Their photocatalytic activity in degrading methyl orange (MO) dye under UV–vis and sunlight irradiation was investigated. The results demonstrated that ILs significantly influenced the structural and optical properties of ZnO, resulting in smaller crystallite sizes, modified morphologies, and reduced band gap energies compared to unmodified ZnO. The ZnO-[BMIM]-BF_4_ (1%) exhibited superior photocatalytic efficiency, achieving complete MO degradation within 30 min under UV–vis irradiation, attributed to its enhanced light absorption and reduced electron–hole recombination. The ZnO-BMIM-PF6 (1%) demonstrated exceptional stability, maintaining high degradation efficiency over multiple cycles. These findings highlight the potential of IL-mediated synthesis in tailoring ZnO nanomaterials for efficient photocatalytic degradation of organic pollutants, offering a promising approach for wastewater treatment.

## 1 Introduction

Dye residues from the textile industry significantly contribute to the contamination of wastewater sources.^1,2^ The textile sector is known for generating large amounts of dye and suspended solid waste.^3^ Alarmingly, it is projected that each year, the environment receives approximately 5000 tons of such dye materials. These harmful substances are known to deplete water’s oxygen content, posing substantial risks to both human health and the broader ecosystem. Moreover, industrial wastewater is fraught with a variety of hazardous compounds, including cyanides, alkaline cleaners, degreasing solvents, and metallic substances, among others.^4^ The escalating problem poses significant threats to both human health and the environment. Industrial wastewater is loaded with a diverse range of harmful elements, including but not limited to cyanides, alkaline detergents, degreasing solutions, oils, fats, and metals.^5−11^

Metal oxide catalysts have emerged as a promising solution for wastewater treatment due to their exceptional photocatalytic properties and ability to degrade various organic pollutants.^12^ Among the numerous metal oxides studied, ZnO, CuO, and NiO have demonstrated significant potential in the photodegradation of contaminants in water. The performance of these catalysts is highly dependent on their synthesis methods, which can influence their shape, particle size, and optical properties.^13−20^

Photocatalysis is a process in which a semiconductor material absorbs light energy to generate electron–hole pairs, initiating redox reactions that degrade organic pollutants into less harmful substances.^14^ For instance, titanium dioxide (TiO_2_)-based photocatalysts have been widely used to degrade dyes like Methyl Orange and pollutants such as phenol in wastewater, as well as in the degradation of emerging contaminants from pharmaceutical pollutants.^21,22^ Additionally, tin dioxide (SnO_2_) nanoparticles have been studied for the photodegradation of Methyl Orange, Methylene Blue, and Rhodamine B dyes.^23^ Zinc oxide (ZnO), which has a similar band gap energy to TiO_2_, has also shown excellent photocatalytic activity in degrading contaminants like Methylene Blue, Rhodamine B, Methyl Orange, and Eriochrome Black T dyes.^24^

Nanosized metal oxide photocatalysts have gained considerable attention in recent years, as they exhibit enhanced degradation efficiency compared to their micro and bulk counterparts.^12^ This improvement is attributed to the quantum confinement effect, which results in unique characteristics for nanosized materials. In addition to wastewater treatment, nanosized metal oxides have found applications in solar cells, fuel cells, gas sensors, hydrogen storage and generation, and antibacterial activities.^12,13,25^ The morphology of metal oxides plays a crucial role in determining their properties and performance.^14,15,26−30^ Researchers have synthesized these materials in various shapes, such as nanospheres, nanowires, nanorods, nanocombs, nanoleaves, and nanobelts. Numerous synthesis techniques have been employed to produce nanostructured metal oxides, including sol–gel, hydrothermal, chemical precipitation, thermal decomposition, and chemical bath deposition methods. The ongoing challenge in this field is to develop single-digit nanometer metal oxide nanostructures with well-defined shapes and sizes, which can further enhance their catalytic efficiency and environmental remediation applications.^31−33^

Ionic liquids (ILs), characterized by their low volatility, high thermal stability, and adaptable solvation, are being increasingly utilized as solvents and reaction media for the creation of a variety of catalysts, as a chemical engineer would appreciate. These unique features enhance the performance of certain catalysts. ILs are capable of forming protective shields on metal nanoparticle (NP) surfaces, which prevent agglomeration, a process facilitated by the establishment of electrical double layers and steric hindrance from the alkyl chains in cations. This interaction ensures the preservation of NP mobility and offers chemical adjustability, which can modify the stability, shape, and electronic properties of the catalysts. Several methods, including chemical reduction, sputtering, and electron beam irradiation, are available for producing metal NPs within ILs. Moreover, employing ILs for electrochemical synthesis/deposition enables the controlled production of NPs, while minimizing the environmental impact.^34−39^

Recent studies have highlighted the significant role of ionic liquids (ILs) in the synthesis and enhancement of semiconductor photocatalysts. Wei et al.^40^ successfully synthesized BiOBr microspheres with oxygen vacancies using ILs. These vacancies effectively capture photogenerated electrons during dye degradation, thereby reducing the recombination rate of electron–hole pairs and enhancing photocatalytic efficiency. In another study, Pascal Voepel and colleagues^41^ prepared polyphase anatase TiO_2_ particle heterojunctions in the presence of ILs, which improved the material’s photocatalytic performance. Similarly, Yang and co-workers^42^ found that IL-assisted synthesis of (BiO)_2_CO_3_ increased the separation rate of photogenerated electron–hole pairs, leading to better photocatalytic activity. Bielicka–Giełdoń et al.^43^ utilized various ILs as halogen sources to prepare bismuth halides. Their findings confirmed that ILs not only relax the crystal structure and decrease particle size but also modify the band gap of bismuth halides. Collectively, these studies demonstrate the significant impact of ILs in the preparation and optimization of semiconductor photocatalysts.

Zinc oxide (ZnO) is one of the most extensively studied photocatalysts due to its affordability, nontoxic nature, and excellent properties. It is widely utilized in various fields, including photocatalytic hydrogen production and the purification of water and air.^44^ Despite ZnO’s relatively high photocatalytic activity, its efficiency remains insufficient for intensive applications. This limitation is primarily attributed to the low separation rate of photogenerated charge carriers.^45,46^ Therefore, enhancing the photocatalytic performance of ZnO by improving the separation of photogenerated electron–hole pairs is crucial.

We have chosen ZnO nanoparticles for this study due to their exceptional photocatalytic properties, which surpass those of many other metal oxides. ZnO has a wide band gap energy (3.37 eV) and a large exciton binding energy (60 meV), making it highly efficient in generating electron–hole pairs under UV irradiation. Additionally, ZnO is abundant, nontoxic, and cost-effective, which are crucial factors for sustainable environmental applications like wastewater treatment.^47−49^

The promise of ionic liquids is considerable and calls for extensive future investigation. In our study, we synthesized and characterized metal oxide nanoparticles in various ionic liquids, investigating their photodegradation efficiency on MO dye. The photocatalysts have been synthesized in in 1-butyl-3-methylimidazolium tetrafluoroborate ([BMIM]-BF_4_), 1-butyl-3-methylimidazolium hexafluorophosphate ([BMIM]-PF_6_), and 1-butyl-3-methylimidazolium chloride ([BMIM]-Cl) liquids.

## 2 Experimental Section

### 2.1 Materials and Methods

All chemicals were sourced from Sigma-Aldrich. Ionic liquids with a purity of >98% were used, including 1-butyl-3-methylimidazolium tetrafluoroborate, 1-butyl-3-methylimidazolium hexafluorophosphate, and 1-butyl-3-methylimidazolium iodide. Other chemicals used were zinc nitrate hexahydrate (Zn(NO_3_)_2_·6H_2_O), and sodium hydroxide (NaOH).

The synthesis of zinc oxide nanoparticles began by dissolving 0.3 M of zinc nitrate in water and adding ionic liquid in percentage volumes of 0.5, 1, and 2%. Vigorous stirring was performed using a magnetic stirrer until a clear, colorless, and transparent solution was achieved. The solution’s pH was then adjusted with NaOH, leading to the formation of a white gel. Stirring continued for an additional 2 h at room temperature, after which the resultant Zn(OH)_2_ sol was allowed to stand for 24 h. The supernatant was discarded, and the settled precursor was recovered via filtration. This was followed by several washings using distilled water and ethanol to eliminate aggregated particles and organic impurities. The cleaned precursor was dried at 80 °C for 12 h, and the resultant Zn(OH)_2_ was then ground. Further heat treatment at 300 °C for 2 h yielded crystallized ZnO nanoparticles.

The same procedure was applied for the synthesis of zinc oxide using different ionic liquids, retaining all the reaction conditions constant. For the sake of comparison, pure zinc oxide was also synthesized using the same protocol, but without the addition of ionic liquids.

### 2.3 Characterization

The specific surface areas of the samples were determined using nitrogen adsorption–desorption isotherm measurements at 77 K (Quantachrome Instrument). Prior to the actual measurements, the samples were degassed at 200 °C for 2 h.^50^

X-ray powder diffraction analysis was performed on the specimens utilizing a Rigaku D/Max-2200 diffractometer, employing Cu Kα radiation with a wavelength (λ) of 1.540 Å. The scanning range for the samples encompassed 10 to 80 degrees 2θ, at a scanning rate of 2 degrees per minute. To determine the sizes of the crystalline domains, the Scherrer equation was applied:
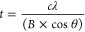
1where λ represents the X-ray wavelength in Ångstroms (Å), *B* corresponds to the full width at half-maximum (fwhm), θ represents the Bragg angle, *C* is a shape-dependent factor (assumed to be unity), and *t* denotes the crystallite size in Ångstroms (Å).^50^

Fourier transform infrared (FTIR) spectra were acquired under ambient conditions using a PerkinElmer Precisely Spectrum One spectrometer with KBr as the diluent. The measurements were conducted at a resolution of 4 cm^–1^, and each spectrum was obtained by averaging 100 scans.

The light absorption properties of the photocatalysts were investigated through UV–vis Ocean optics model DH-2000-BAL spectrophotometer. The spectra results were recorded in the 280–1000 nm range using halogen and deuterium lamps as light sources. and the absorption band gap energy (Eg) was determined utilizing the Kubelka–Munk function.^51^ SEM images of the photocatalyst samples were acquired at various magnifications using a Zeiss EVO MA10 SEM instrument (Germany), operating at an acceleration voltage of 10 kV.^52^

Photoluminescence (PL) spectra were acquired at room temperature using a PerkinElmer LS-50 fluorescence spectrophotometer. The sample was first dispersed in ethanol via ultrasonication. A xenon lamp served as the excitation source, and an excitation wavelength of 325 nm was used for all measurements.^50^

The photocatalytic performance of the catalysts was assessed through the degradation of methyl orange. To conduct these experiments, we utilized the LUZCHEM LZC-5 photoreactor system equipped with UV lamps, specifically 64 W UV–B, 64 W UV–A, and 64 and 100 W halogen lamps as light sources. In a typical experimental procedure, 100 mg of the catalyst was dispersed in 50 mL of aqueous dye solutions with an initial concentration of 25 mg/L and maintained at a neutral pH. This mixture was subjected to magnetic stirring after undergoing 10 min of ultrasonication to ensure the establishment of an adsorption–desorption equilibrium between the catalyst and the solution. This step was performed in the dark. Subsequently, the aqueous dye solution was exposed to different time intervals of illumination over a span of 2 h. After the photocatalytic treatment, the solution was filtered using a membrane filter with a pore size of 0.45 μm to separate the catalyst from the dye solution. The filtered solution was then further analyzed using UV–vis absorption spectroscopy.^53,54^

## 3 Results and Discussion

### 3.1 Structural and Optical Properties of the Photocatalyst

The specific surface areas of the catalysts after heat treatment at 200 °C were measured using the Brunauer–Emmett–Teller (BET) method with N_2_ adsorption. As detailed in Table 1, the specific surface area (SBET) varied depending on the metal oxide modification. The SBET values for ZnO, ZnO-[BMIM]-BF4(1%), ZnO-BMIM-Cl(1%), ZnO-BMIM-PF6(1%), ZnO-BMIM-BF4(0.5%), and ZnO-BMIM-BF4(2%) were 29, 20, 19, 21, 19, and 15 m^2^/g, respectively. The generally lower specific surface area observed for the modified catalysts compared to unmodified ZnO can be attributed to the adsorption of ionic liquid (IL) molecules onto the ZnO surface. This interaction may partially block surface pores, thereby reducing the effective surface area available for gas adsorption during the BET analysis, as reported in.^55^ However, it is important to note that photocatalytic activity is not solely determined by a high BET surface area. The quantity and distribution of active sites play a more significant role in catalytic reactions than a large surface area.

**Table 1:** Surface and Optical Results of Photocatalysts, Pseudo-First-Order Kinetic Parameters, and Efficiency of MO Degradation under UV–B Irradiation after 60 min

catalysts	crystallite size (nm)	band gap (eV)	SBET (m^2^ g^–1^)	methyl orange degradation efficiencies (%)	*k*_r_ (min^–1^)	*R*^2^
ZnO	64	3.25	29	75	0.02	0.99
ZnO-[BMIM]-BF4(1%)	28	2.50	20	100	0.16	0.98
ZnO-BMIM-Cl(1%)	29	2.57	19	90	0.036	0.99
ZnO-BMIM-PF6(1%)	31	2.58	21	97	0.111	0.998
ZnO-BMIM-BF4(0.5%)	33	2.65	19	100	0.067	0.999
ZnO-BMIM-BF4(2%)	32	2.60	15	88	0.036	0.993

Despite the reduced specific surface area, the enhanced photocatalytic performance of IL-modified ZnO suggests that the adsorption of IL molecules does not significantly impede the adsorption of MO. This indicates that the beneficial effects of IL modification, such as improved charge separation and increased generation of reactive species, outweigh any potential blocking of adsorption sites.

**Figure 1:**
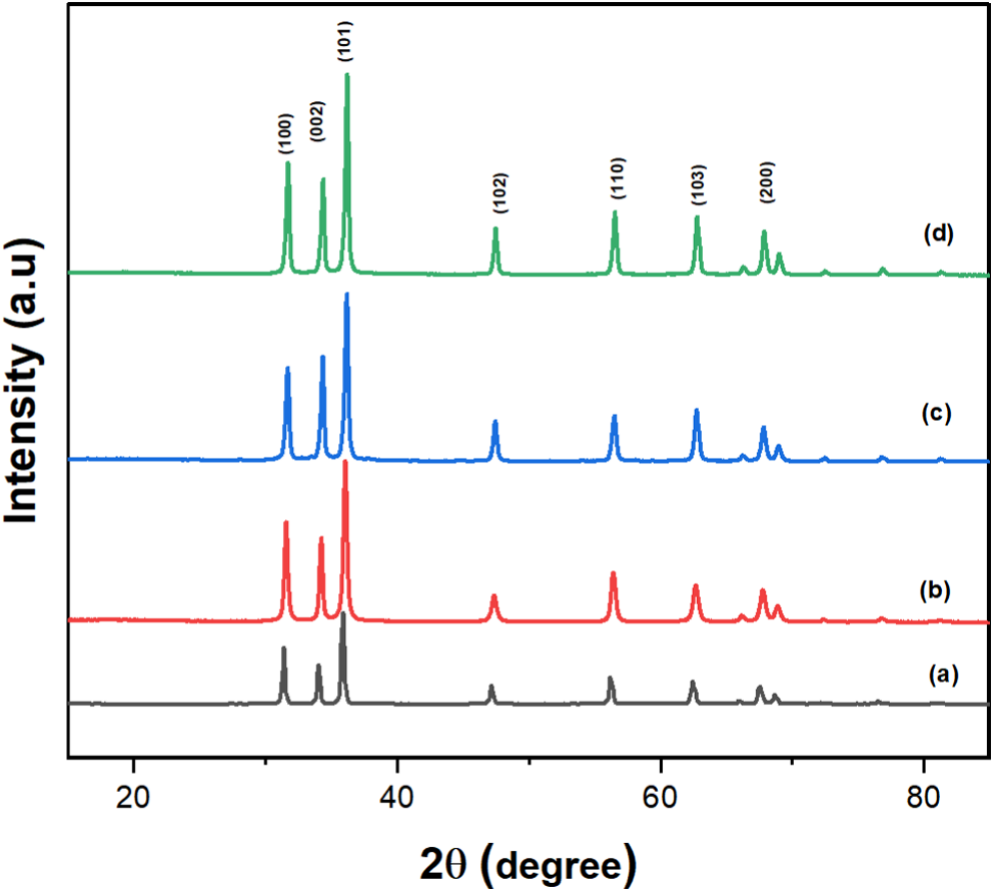
XRD of synthesized: (a) Pure ZnO nanoparticles and ZnO nanoparticles in (b) [BMIM]BF_4_ (%1), (c) [BMIM]Cl (1%), and (d) [BMIM]PF_6_ (1%).

**Figure 2:**
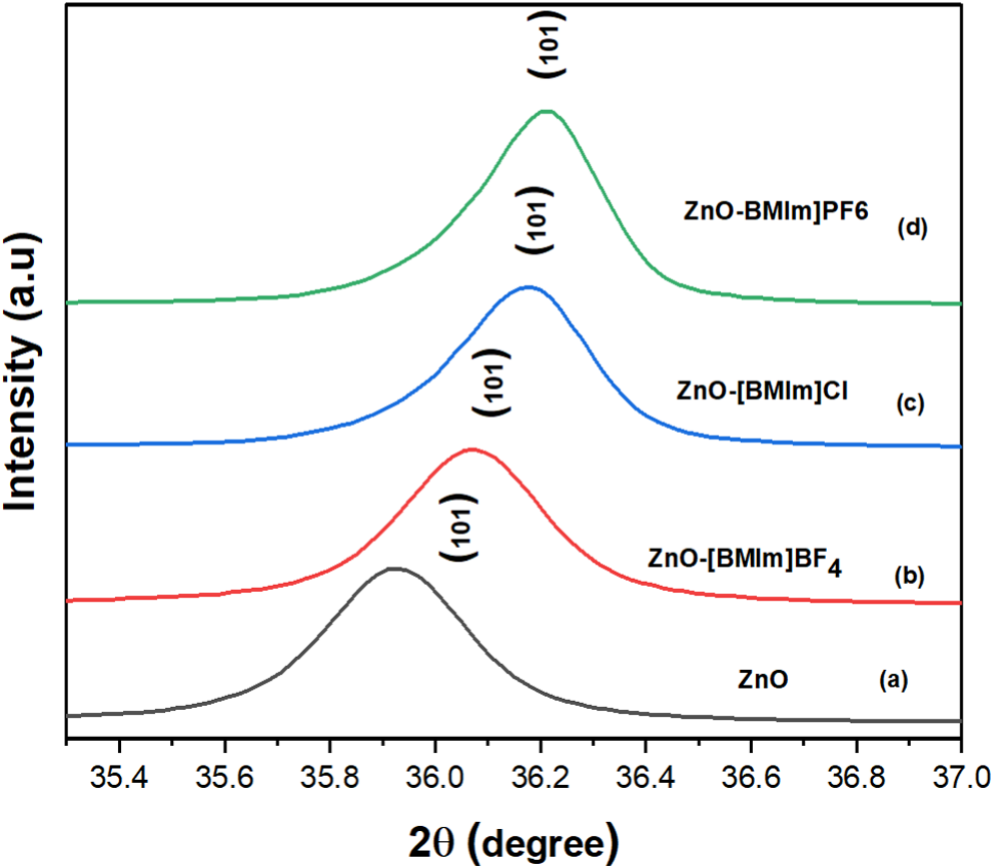
Analysis of the (101) peak shifts of (a) Pure ZnO nanoparticles and ZnO nanoparticles in (b) [BMIM]BF_4_ (%1), (c) [BMIM]Cl (1%), and (d) [BMIM]PF_6_ (1%).

**Figure 3:**
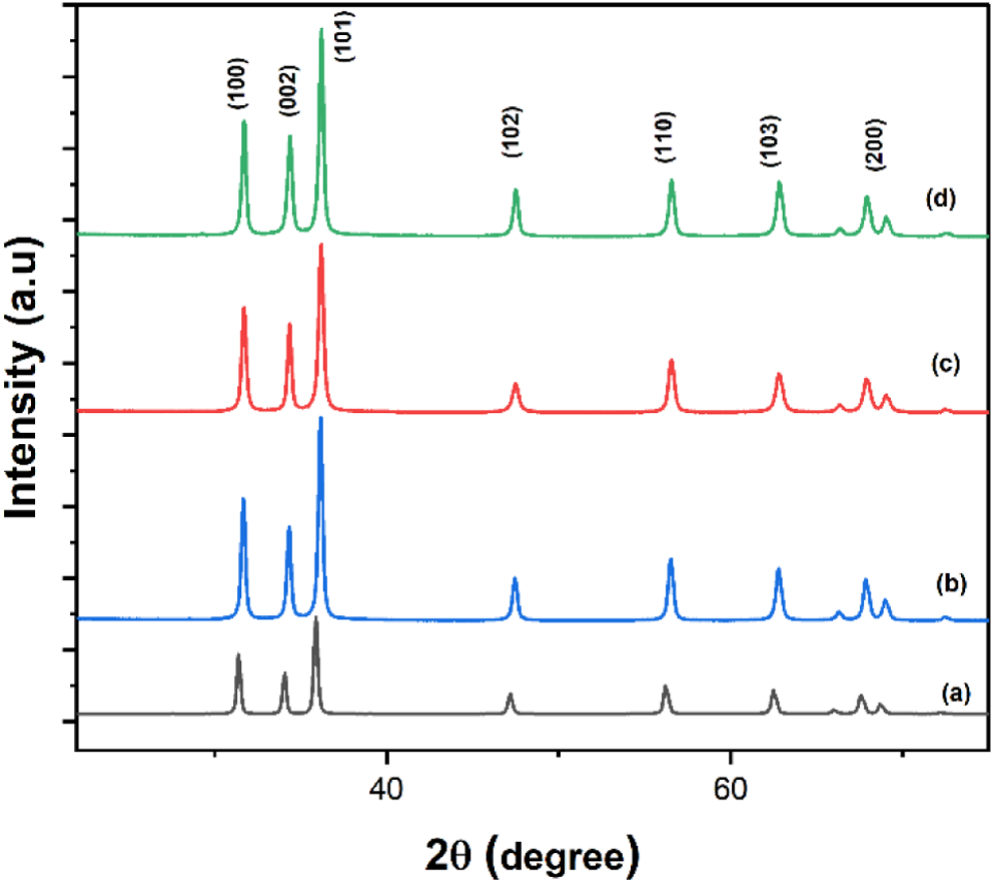
XRD of synthesized: (a) Pure ZnO nanoparticles and ZnO nanoparticles in (b) [BMIm]BF_4_ (0.5%), (c) [BMIM]BF_4_ (1%), and (d) [BMIm]BF_4_ (2%).

**Figure 4:**
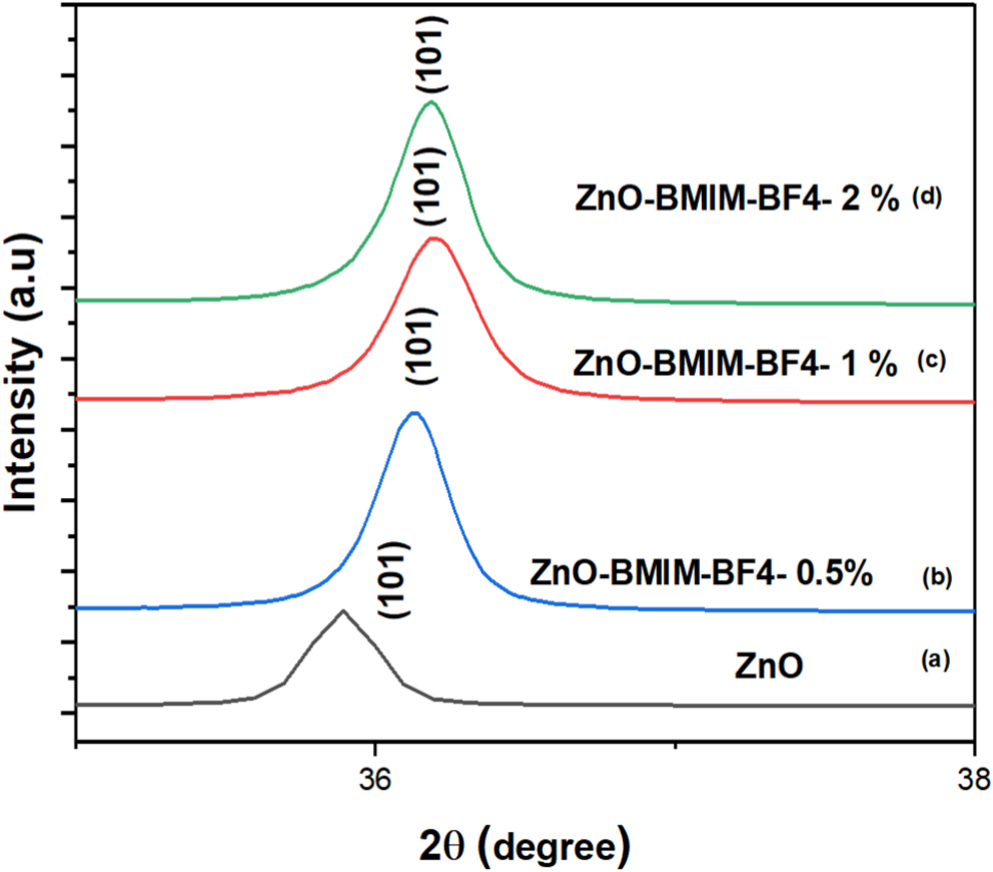
Analysis of the (101) peak shifts of (a) Pure ZnO nanoparticles and ZnO nanoparticles in (b) [BMIm]BF_4_ (0.5%), (c) [BMIM]BF_4_ (1%), and (d) [BMIm]BF_4_ (2%).

X-ray diffraction (XRD) analysis was employed to investigate the phase, purity, and crystallite size of the synthesized ZnO nanomaterials. This analysis was performed on pure ZnO-NP and ZnO-IL as shown in Figures 1 and 2. Further investigation focused on ZnO photocatalysts prepared with varying volume percentages of [BMIM]-BF_4_ as shown in Figures 3 and 4. The XRD patterns presented in Figures 2 and 4 show that the peaks are shifted to higher 2θ values due to the addition of ionic liquids, indicating changes in the crystal lattice parameters and confirming the presence of highly crystalline ZnO structures exhibiting a hexagonal wurtzite configuration (JCPDS card No. 36-1451), aligning with findings reported in.^56,57^ The XRD patterns in Figures 1 and 3 clearly demonstrate the complete conversion of the Zn(OH)_2_ precursor into the hexagonal wurtzite phase of ZnO, regardless of the ionic liquid used. Importantly, no significant peaks were observed that could be attributed to other ZnO phases or impurities, highlighting the high purity of the synthesized materials. The sharp and well-defined diffraction peaks further underscore the high crystallinity of the ZnO nanostructures. Interestingly, Figures 2 and 4 illustrate a shift in the XRD pattern toward higher 2θ angles. This shift indicates alterations in the ZnO crystal lattice, potentially caused by factors such as strain, defects, or thermal expansion induced by the ionic liquid environment during synthesis. This observation is consistent with findings reported in.^58,59^ The Debye–Scherrer equation^58^ was employed to determine the average crystallite size of the synthesized ZnO nanomaterials as shown in Table 1. Notably, ZnO synthesized using [BMIM]-BF_4_ ionic liquid exhibited the smallest crystallite size (28 nm) and the highest degree of crystallinity compared to ZnO-[BMIM]-Cl (29 nm), ZnO-[BMIM]-PF_6_ (31 nm), ZnO-[BMIM]-BF_4_ (0.5%) (33 nm), ZnO-[BMIM]-BF_4_ (2%) (32 nm), and pure ZnO (64 nm). This smaller crystallite size contributes to a larger surface area with more active sites, explaining the superior dye removal ability observed for ZnO-[BMIM]-BF_4_(1%).^60,61^ Furthermore, the relative intensities of the diffraction peaks suggest preferential growth along specific crystallographic planes, particularly the (101) plane, influenced by the presence of ionic liquids.^61,62^ Additionally, the variations in relative intensities of diffraction peaks among samples synthesized with different ionic liquids indicate that each ionic liquid uniquely influences the directional growth of the nanostructures. In conclusion, XRD analysis confirmed the successful synthesis of phase-pure ZnO nanomaterials possessing well-defined crystal structures and controlled crystallite sizes. The incorporation of ionic liquids during synthesis was found to induce changes in the crystal lattice, likely due to strain, defects, or thermal expansion. These findings highlight the potential of utilizing ionic liquids for tailoring the structural properties of ZnO nanomaterials for specific applications.

**Figure 5:**
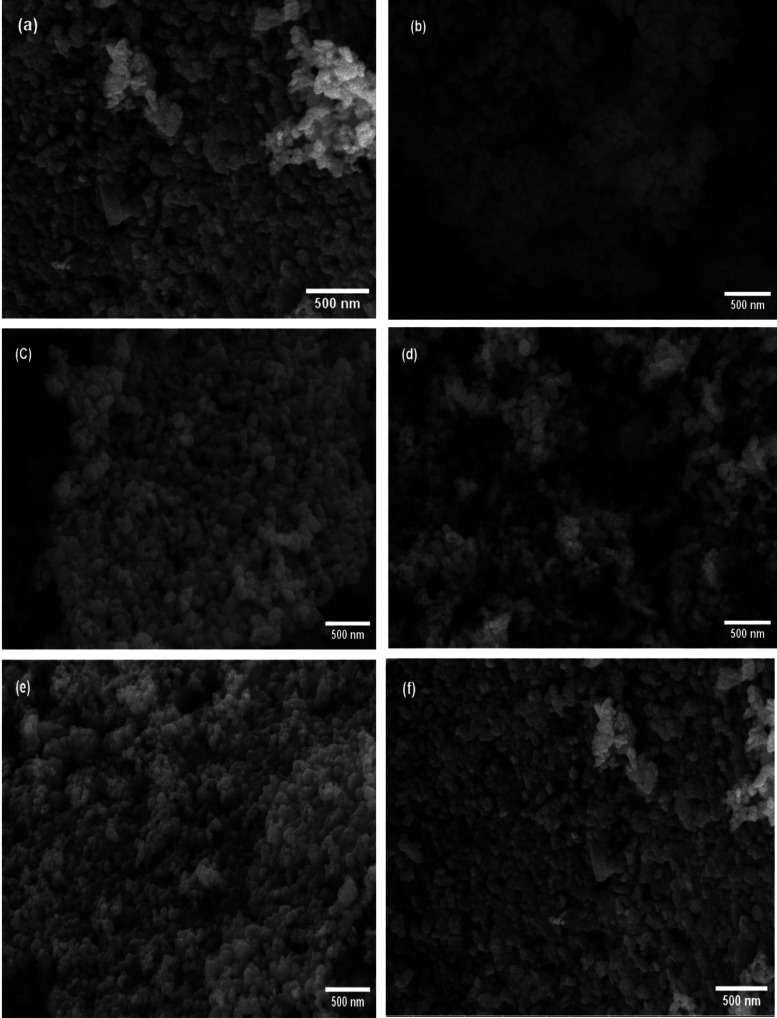
SEM images of synthesized: (a) Pure ZnO nanoparticles and ZnO nanoparticles in (b) [BMIm]BF_4_ (1%), (c) [BMIm]Cl (1%), (d) [BMIm] PF_6_ (1%), (e) [BMIM]-BF_4_ (0.5%), (f) [BMIM]-BF_4_ (2%). Magnification is 100,000X for a scale bar of 500 nm.

The SEM images in Figure 5a–f display the morphologies and dispersity of nanostructured pure ZnO nanoparticles and ZnO photocatalysts synthesized in three unique ionic liquids. These images reveal well-defined ZnO nanostructures, composed of nanosized particles that are regular and uniformly shaped. In Figure 5a, the SEM image of pure ZnO shows capsule-shaped particles with an average size of approximately 105 nm. Figure 5b presents ZnO-[BMIM]-BF_4_, which also exhibits capsule structures but with a smaller average particle size of about 35 nm. Figure 5c shows ZnO prepared from [BMIM]-Cl, with a mean diameter of around 65 nm. These ZnO particles exhibit a capsule-shaped morphology without any agglomeration. The morphology and size of ZnO-[BMIM]-PF_6_, depicted in Figure 5d, reveal capsule-like particles with an average size of approximately 78 nm. This size is larger than that of ZnO-[BMIM]-BF_4_, likely due to the shorter alkyl moiety of the countercation when the highly coordinated anion PF_6_ is used instead of BF_4_ and Cl.^63^Figures 5e,f show SEM images of ZnO-[BMIM]-BF_4_ synthesized at different concentrations, 0.5 and 2%, respectively. Both display capsule-shaped morphologies with average particle sizes of approximately 48 and 60 nm, respectively. The different anions likely interact differently with the [BMIM] cation or the solvent, leading to variations in aggregation and consequently, particle size. SEM analysis visually confirms the well-defined and uniformly shaped ZnO nanostructures, emphasizing the role of distinct ionic liquids in controlling particle morphology. The absence of agglomeration and aggregation further highlights the potential of ionic liquids as effective modifiers for enhancing the photocatalytic performance of metal oxide semiconductors.

**Figure 6:**
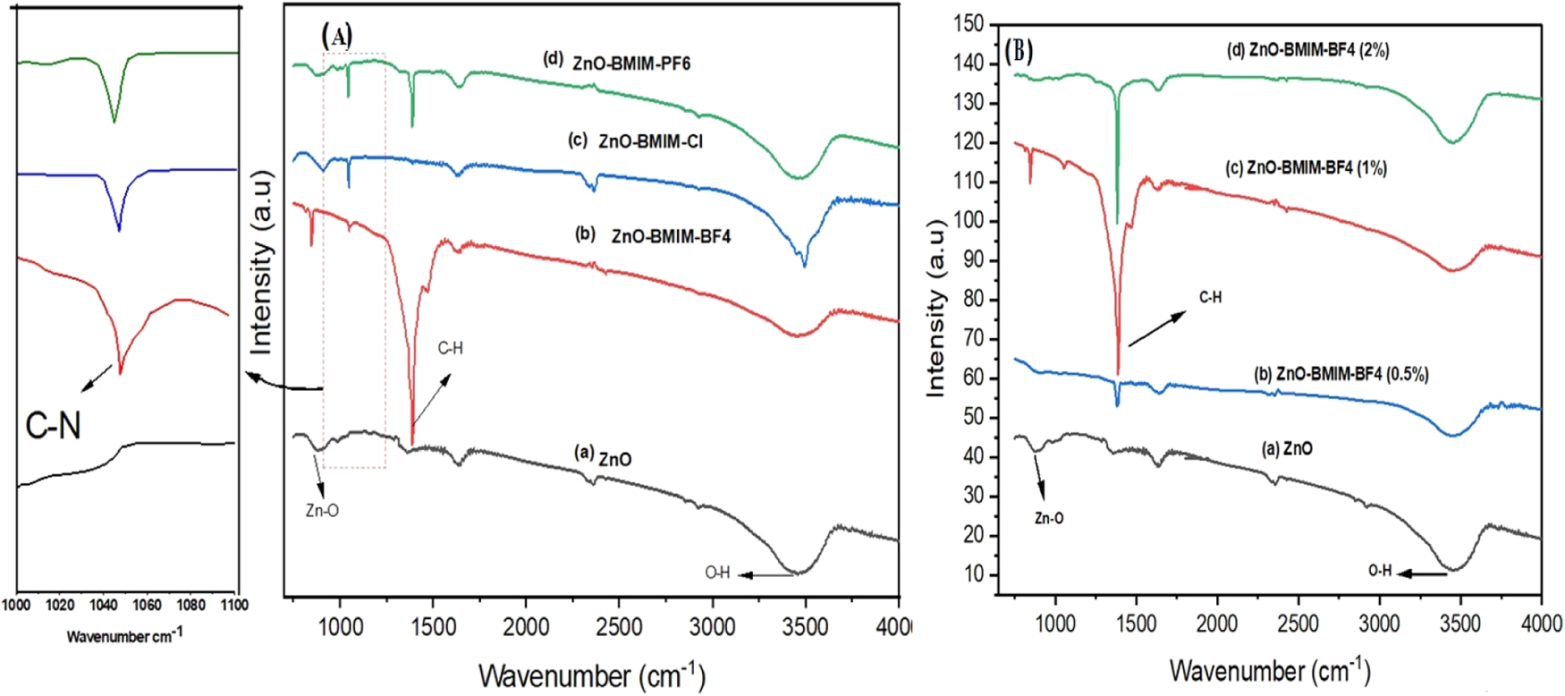
(A) FT-IR spectra of synthesized ZnO nanoparticles: (a) pure ZnO, (b) ZnO in [BMIM]-BF_4_ (1%), (c) ZnO in [BMIM]-Cl (1%), and (d) ZnO in [BMIM]-PF_6_ (1%). (B) FT-IR spectra of synthesized ZnO nanoparticles at varying [BMIM]-BF_4_ concentrations: (a) pure ZnO, (b) ZnO in [BMIM]-BF_4_ (0.5%), (c) ZnO in [BMIM]-BF_4_ (1%), and (d) ZnO in [BMIM]-BF_4_ (2%).

Fourier-transform infrared (FT-IR) spectroscopy was employed to characterize the structural composition of the synthesized photocatalyst samples. Figure 6a presents the FT-IR spectra of pure ZnO-NP and ZnO-IL photocatalysts. Figure 6b focuses on the FT-IR spectra of ZnO photocatalysts synthesized using varying concentrations of [BMIM]-BF4. The spectra in Figure 6a,b reveal distinct peaks at 519 and 1423 cm^–1^ for all ZnO samples. These peaks correspond to the stretching vibrations of the Zn–O bond and the C–H bonds, respectively.^54,64^ Furthermore, a peak at 3424 cm^–1^, attributed to hydroxyl groups (O–Hstr.) associated with ZnO nanoparticles (ZnONPs), confirms their successful formation. Figure 6a also presents the C–N stretching vibration at 1055 cm^–1^, which is attributed to the presence of nitrogen resulting from the interaction between the ionic liquid and ZnO.^65,66^ Furthermore, the ZnO sample modified with [BMIM]-BF4 (1%) exhibits the highest intensity for the C–H stretching vibration peak compared to samples modified with other ionic liquids. This suggests a stronger interaction between the [BMIM]-BF4 (1%) and the ZnO, potentially due to a higher concentration of C–H bonds within this ionic liquid or a more favorable interaction with the ZnO surface.^55,67^ Examining Figure 6b, where curve (a) represents pure ZnO, we observe characteristic peaks associated with Zn–O and O–H bonds. Curves (b), (c), and (d) represent ZnO-[BMIM]-BF4 at increasing concentrations (0.5, 1, and 2% respectively). Each subsequent addition of [BMIM]-BF4 leads to noticeable shifts in peak intensities and positions, indicating the ionic liquid’s influence on the vibrational properties of ZnO. This suggests that the [BMIM]-BF4 ionic liquid plays a significant role in altering the chemical structure of the synthesized ZnO. The observed FT-IR signatures suggest that the employed ionic liquids actively participate in the metal oxide network formation during synthesis. This involvement potentially facilitates the creation of novel organometallic complexes, as reported in ref (67).^67^

**Figure 7:**
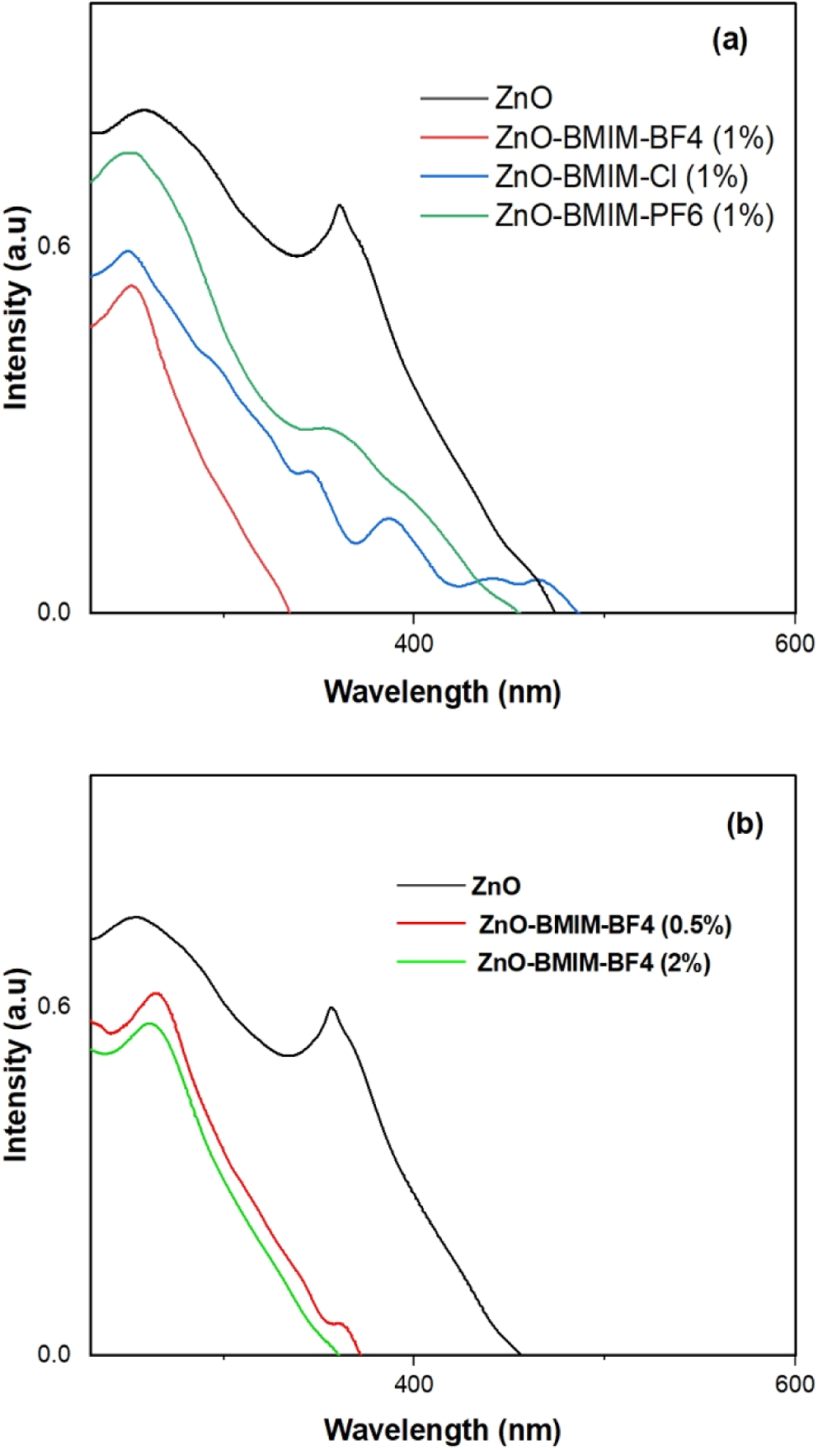
(a) and (b) UV–Vis absorption spectra of pure ZnO nanoparticles and ZnO photocatalysts synthesized in various ionic liquids.

The absorption edges of the photocatalyst samples exhibit a progressive red shift with the addition of ionic liquids, as illustrated in Figure 7a,b. The absorption edge shifts from 365 nm for pure ZnO to 250 nm for ZnO-[BMIM]-BF4(1%), demonstrating the influence of ionic liquids on the electronic structure. Specifically, the absorption edges are located at 260, 265, 258, and 257 nm for ZnO-BMIM-BF4(2%), ZnO-BMIM-BF4(0.5%), ZnO-BMIM-PF6(1%), and ZnO-BMIM-Cl(1%), respectively. This red shift suggests modifications in the electronic transitions or band structures of ZnO due to the presence of ionic liquids, as evidenced by the UV–visible spectra in Figure 7a–c. Notably, [BMIM]-BF4 (1%) results in the smallest wavelength, indicating the most significant impact on the absorption edge.

Furthermore, increasing the volume percentage of [BMIM]-BF4 from 0.5 to 2% leads to a decrease in wavelength, with 1% emerging as the optimal concentration within the studied range. These observations highlight the potential of ionic liquids, particularly [BMIM]-BF4, as promising modifiers for enhancing the photocatalytic performance of metal oxide semiconductors. The blue-shifted absorption edges (<400 nm), attributed to the zinc crystal structure of the solid solution, further support this conclusion.

**Figure 8:**
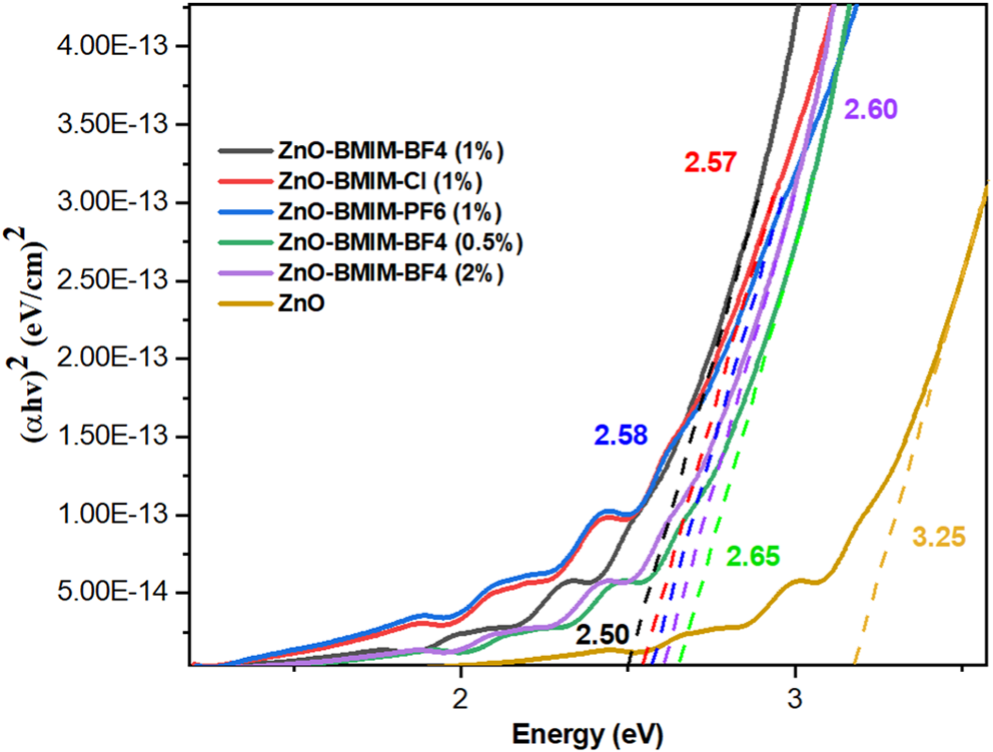
Tauc plots of pure ZnO nanoparticles and ZnO photocatalysts synthesized in different ionic liquids.

The band gaps of the synthesized photocatalysts were determined using Tauc line plots, where (αhυ)^2^ was plotted against the energy of absorbed light (Figure 8). In this context, α represents the absorption coefficient and hυ denotes the discrete photon energy. The absorption edge energies were obtained by extrapolating the linear portion of the Tauc plots to α = 0, utilizing the Kubelka–Munk equation transformation.

The calculated band gap energies for the ZnO photocatalysts are presented in Table 1. Notably, ZnO-[BMIM]-BF4 (1%) exhibited the lowest band gap energy of 2.50 eV, indicating the optimal concentration within the group of ZnO photocatalysts modified with [BMIM]-BF4. Increasing the volume percentage of [BMIM]-BF4 from 0.5 to 2% led to a decrease in the band gap energy from 2.65 to 2.50 eV. This reduction in band gap energy, compared to the higher value of 3.25 eV observed for other ZnO photocatalysts, highlights the significant impact of ionic liquid-assisted synthesis on the photocatalytic material, a phenomenon also reported in previous studies.^68−70^

The determined band gap values for all ZnO-[BMIM]-X nanostructures, ranging from 2.50 to 3.25 eV, are sufficiently large to effectively drive the photodegradation of organic dyes. This characteristic makes them advantageous for utilizing a broader range of sunlight irradiation, including the visible region.

The absorption edge shifted from around 365 nm for pure ZnO to approximately 380 nm for the composites, indicating enhanced absorption in the visible region. Correspondingly, the band gap energy decreased from 3.25 eV for pure ZnO to 2.50 eV for ZnO-[BMIM]-BF_4_ (1%). This reduction is attributed to the effect of [BMIM]-BF_4_ on the ZnO nanoparticles during synthesis. The ionic liquid may introduce defects, such as oxygen vacancies or interstitial zinc, which create new energy levels within the band gap. These modifications facilitate the absorption of lower-energy photons, thus reducing the band gap and improving photocatalytic efficiency under visible light.^71−73^

PL emission spectra provide insights into the efficiency of charge carrier trapping, migration, and transfer processes within semiconductor particles. As PL primarily arises from the recombination of excited electrons and holes, a lower PL intensity generally indicates a reduced electron–hole recombination rate under illumination.^50^

**Figure 9:**
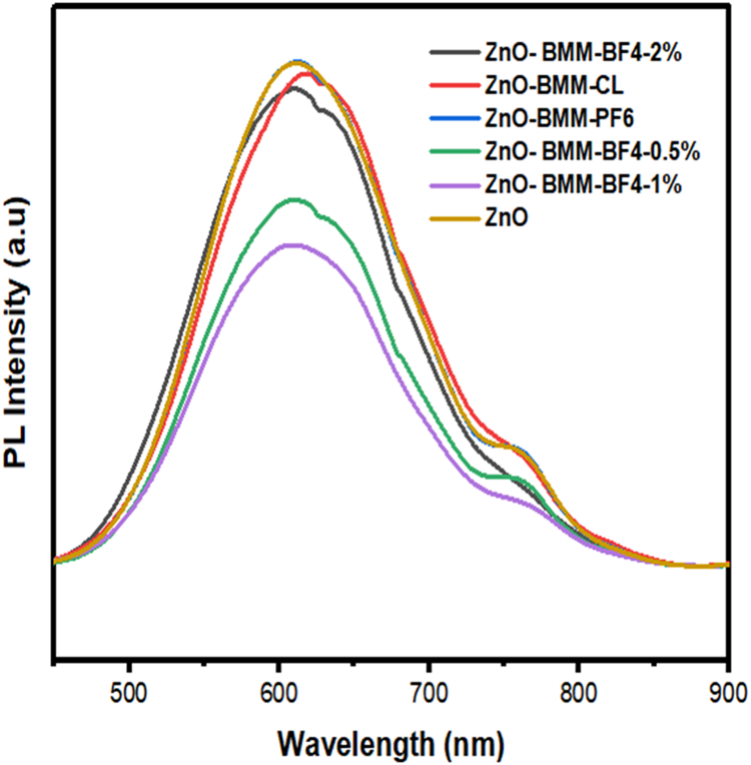
PL spectra of pure ZnO nanoparticles and ZnO photocatalysts synthesized in different ionic liquids.

Figure 9 presents the PL spectra of the prepared photocatalysts under an excitation wavelength of 600 nm. All photocatalysts exhibit main emission peaks at similar positions, albeit with varying intensities. Notably, ZnO-BMIM-BF4 (0.5%) and ZnO-BMIM-PF6 (1%) display the lowest relative PL intensities, suggesting a reduced electron–hole recombination rate. In contrast, ZnO-BMIM-BF4 (2%), ZnO-BMIM-PF6 (1%), and ZnO-BMIM-Cl (1%) exhibit intensities comparable to that of pure ZnO, implying a higher probability of electron–hole recombination in these samples.

The suppressed PL intensity observed for ZnO-BMIM-BF4 (0.5 and 1%) suggests that the addition of BMMB-F4 at these concentrations effectively inhibits electron–hole recombination, potentially enhancing photocatalytic activity, this finding aligns with^50^ report. Notably, the ZnO-BMIM-PF6 (1%) catalyst exhibits the lowest PL intensity among all synthesized materials, indicating the most effective suppression of electron–hole recombination, which correlates well with its photocatalytic activity results.

### 3.2 Degradation Studies

The presented Table 1 shows photocatalysts based on pure ZnO-NP and ZnO-IL photocatalysts, which were synthesized in three unique ionic liquids ([BMIM]-BF4, [BMIM]-PF_6_, and [BMIM]-Cl), and compares them to their unmodified counterparts. The band gap, a crucial parameter for light absorption, ranges from 2.5 to 3.25 eV. The table also displays the degradation efficiencies of MO dye under UV–visible light irradiation for the different catalysts. All modified ZnO catalysts demonstrate higher degradation efficiencies than unmodified ZnO, with ZnO-[BMIM]-PF4 achieving the highest efficiency of 100% in 60 min. The kinetic equation is shown in eq 2.
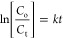
2Where: *C*_o_ is the initial concentration, *C*_t_ is the MO concentration at time, and *k* (min^–1^) is the rate constant of the observed pseudo-first order reaction. To compare the activities of the catalysts, the photocatalytic degradation efficiencies and reaction rates were calculated and the activity values are given in the table. The rate constants (*k*), with ZnO-[BMIM]-PF6 recording the highest value (0.111 min^–1^). These results imply that the ionic liquids contribute to enhancing the photocatalytic activity of ZnO in MO degradation.

**Figure 10:**
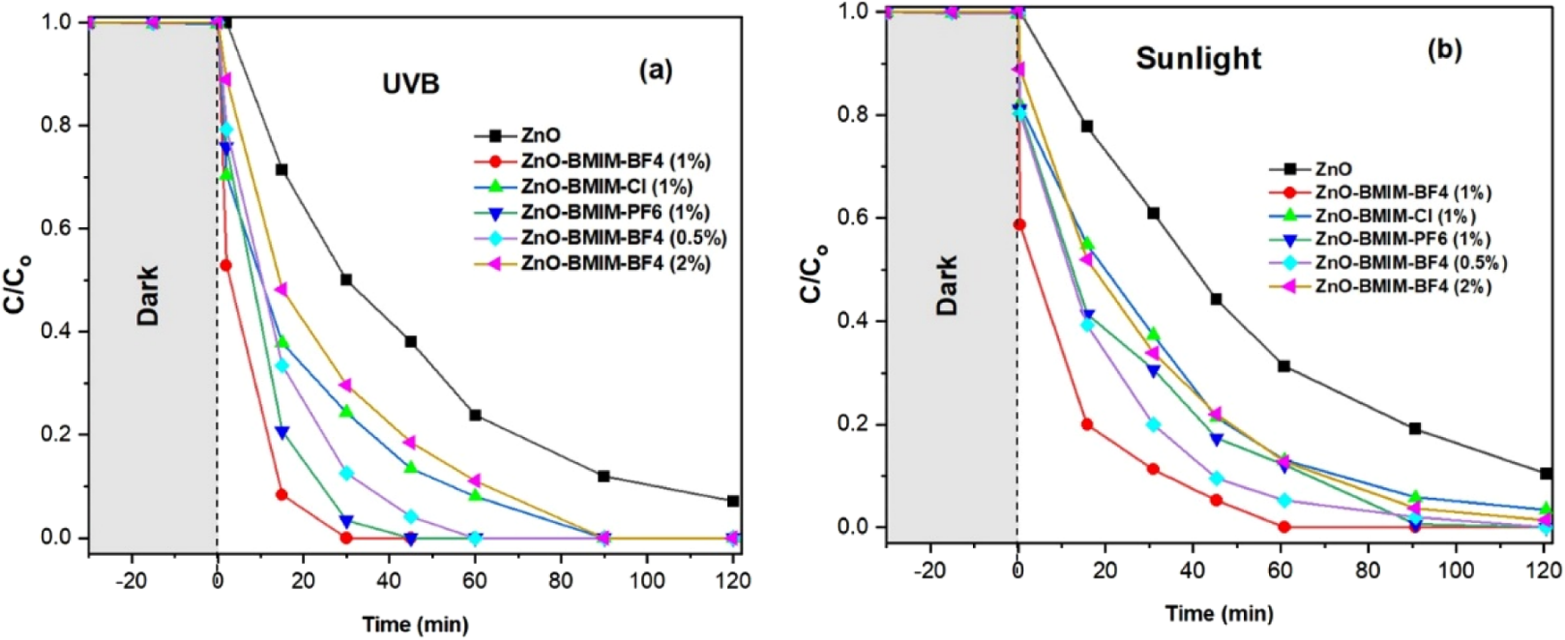
(a) Photodegradation of methyl orange (MO) under UV–B irradiation by pure ZnO nanoparticles and ZnO photocatalysts synthesized in different ionic liquids. (b) Photodegradation of methyl orange (MO) under sunlight irradiation by pure ZnO nanoparticles and ZnO photocatalysts synthesized in different ionic liquids.

Figure 10a,b illustrates the photocatalytic degradation of MO under both UV–B and sunlight irradiation. The figure compares the performance of pure ZnO nanoparticles against ZnO photocatalysts modified with various ionic liquids ([BMIM]-BF4, [BMIM]-Cl, and [BMIM]-PF6).

As shown in Figure 10a, the photocatalytic efficiencies, calculated based on the removal of MO after 60 min of irradiation, follow a distinct trend. ZnO-[BMIM]-BF4 (1 and 0.5%) exhibited the highest degradation rate, achieving complete (100%) removal of MO. Notably, in the presence of ZnO-[BMIM]-BF4 (1%), complete degradation was achieved within just 30 min. Following this, ZnO-BMIM-PF6 demonstrated a degradation rate of 96.5%, while increasing the [BMIM]-BF4 concentration to 2% in ZnO-[BMIM]-BF4–2% resulted in a slightly lower degradation rate of 88%. ZnO-[BMIM]-CL and pure ZnO showed comparatively lower degradation rates of 90 and 75%, respectively.

Similar trends are observed under sunlight irradiation as shown in Figure 10b. ZnO-[BMIM]-BF4 (1 and 0.5%) again demonstrated the highest degradation rates, achieving 100% removal of MO within 60 min. ZnO-BMIM-PF6 showed a degradation rate of 87.5%, while ZnO-[BMIM]-BF4–2%, ZnO-[BMIM]-CL, and pure ZnO exhibited lower rates of 86, 70, and 40%, respectively.

Importantly, the photodegradation of MO under dark conditions (no irradiation) was negligible for all photocatalysts, ranging from 0.01 to 0.05%. This highlights the crucial role of light irradiation in driving the photocatalytic degradation process.

Moreover, the results of a degradation experiment conducted under three different conditions: in the dark, under sunlight irradiation, and under UV–vis irradiation, all without the presence of a photocatalyst. Results show that MO exhibited negligible degradation in the absence of a photocatalyst, even after 90 min of exposure to sunlight or UV–vis irradiation. Specifically, no degradation was observed under dark or sunlight conditions, and only a negligible degradation of approximately 0.01% was observed under UV–B irradiation. These findings underscore the essential role of a photocatalyst in initiating and accelerating the degradation process for MO.

This data highlights that modifying ZnO with [BMIM]-BF4 significantly enhances its photocatalytic activity, surpassing the performance of other tested ionic liquids. The superior performance of ZnO-[BMIM]-BF4 can be attributed to The enhanced degradation rate can be attributed to the ability of ionic liquids (ILs) to generate reactive species and facilitate the efficient transfer of photogenerated electrons and holes to the catalyst surface, thereby accelerating the degradation process.^69,70^

Recent advancements in the field of photocatalytic degradation of MO have been noteworthy. Regraguy et al. demonstrated improved efficiency in photocatalytic degradation using NiSO_4_/TiO_2_ nanoparticles, resulting in a 0.1% increase in degradation efficacy.^74^ Another study introduced a novel organic composite photocatalyst synthesized via the sol–gel method, showing promising results in UV light-induced degradation of MO, achieving complete removal (100%) within 120 min.^75^

Notably, Sadegh et al.^76^ demonstrated the effectiveness of Fe_3_O_4_–CuS/SiO_2_ as a photocatalyst for visible light-assisted degradation of methyl orange (MO), achieving a remarkable 78% degradation within 20 min. Meanwhile, Kader et al.^77^ explored the potential of MoO_3_ and Ag-doped TiO_2_ photocatalysts for enhancing the photodegradation of MO under UV irradiation. Their findings showed that Ag/MoO_3_/TiO_2_ photocatalysts led to a 75.8% degradation rate after 5.5 h of UV irradiation. More recent research in 2024^78^ focused on the synthesis of Ag/AgI/Ag_3_VO_4_ hybrid photocatalysts through ion exchange techniques, achieving an impressive 94.4% degradation of MO within just 15 min.

Moreover, Makota et al. modified Fe_3_O_4_@SiO_2_@ZnO composite, exhibiting enhanced photocatalytic activity and achieving a 96% removal of MO in 240 min during photodegradation under UV irradiation.^79^ These studies collectively highlight ongoing efforts to enhance the efficiency and efficacy of photocatalytic processes in MO degradation.

In conclusion, the results presented in Table 1, and Figures 7 and 10 demonstrate that modifying ZnO with ionic liquids can significantly enhance its photocatalytic activity toward the degradation of organic dyes under UV–B light irradiation. The choice of ionic liquid significantly influences the photocatalytic activity, with [BMIM]-BF4 (1%) generally yielding the highest efficiencies for ZnO photocatalysts. These findings highlight the potential of ionic liquids as promising modifiers for enhancing the performance of metal oxide semiconductors in photocatalytic applications.

**Figure 11:**
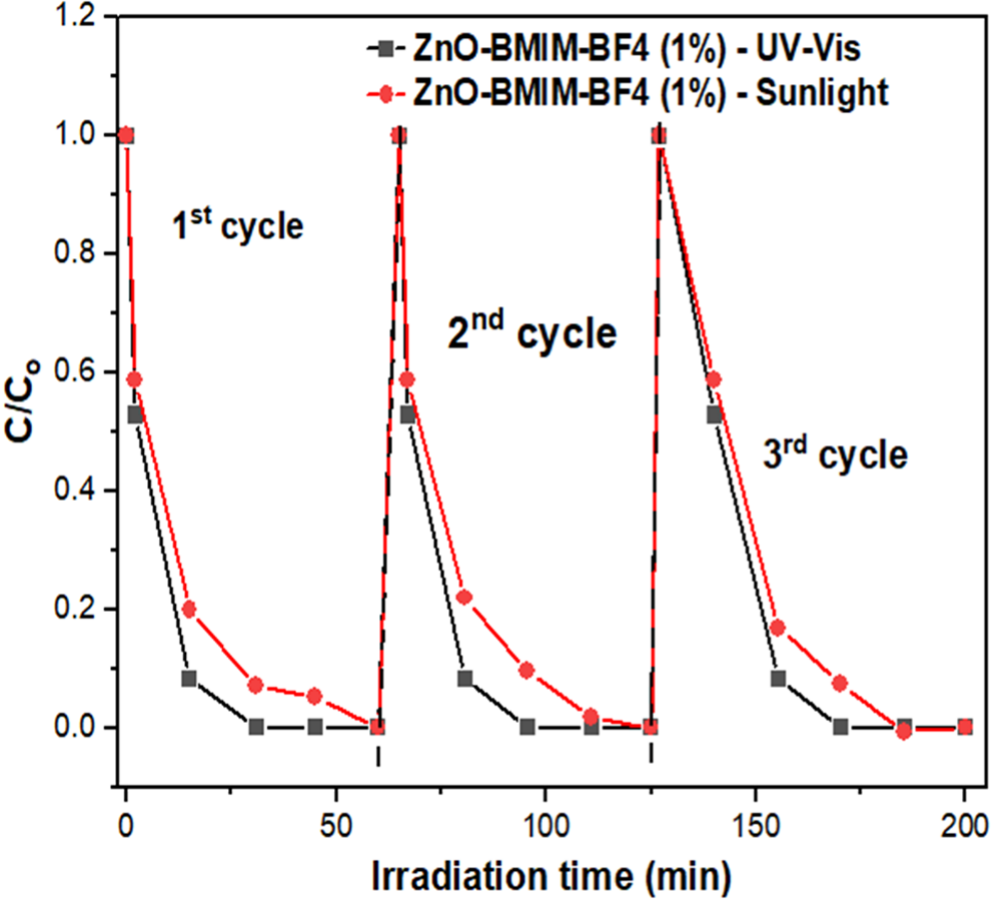
Stability tests for the photocatalytic degradation under sunlight and UV–B irradiation of MO on ZnO-BMIM-PF6(1%) photocatalyst.

Figure 11 illustrates the remarkable stability of the ZnO-BMIM-PF6 (1%) photocatalyst during the photocatalytic degradation of MO. The experiment involved multiple cycles of degradation under both sunlight and UV–B irradiation. Significantly, the catalyst maintained its high performance across all three cycles, achieving complete degradation of MO within 30 min under UV–B light and 60 min under sunlight. This consistent and rapid degradation over multiple cycles underscores the potential of ZnO-BMIM-PF6 (1%) as a robust and reusable photocatalyst for environmental remediation applications.

Compared to traditional synthesis techniques, the IL-assisted approach offers enhanced control over the nucleation and growth processes of ZnO nanoparticles. The unique properties of ILs, such as their ability to stabilize specific crystal facets and prevent agglomeration, lead to the formation of nanoparticles with smaller sizes, uniform morphologies, and improved optical properties. These enhancements are critical for optimizing photocatalytic performance, thereby demonstrating the novelty and effectiveness of the IL-assisted synthesis in producing superior ZnO nanocatalysts.

### 3.3 Proposed Mechanism of Photocatalyst Formation in the Presence of Ionic Liquids

**Figure 12:**
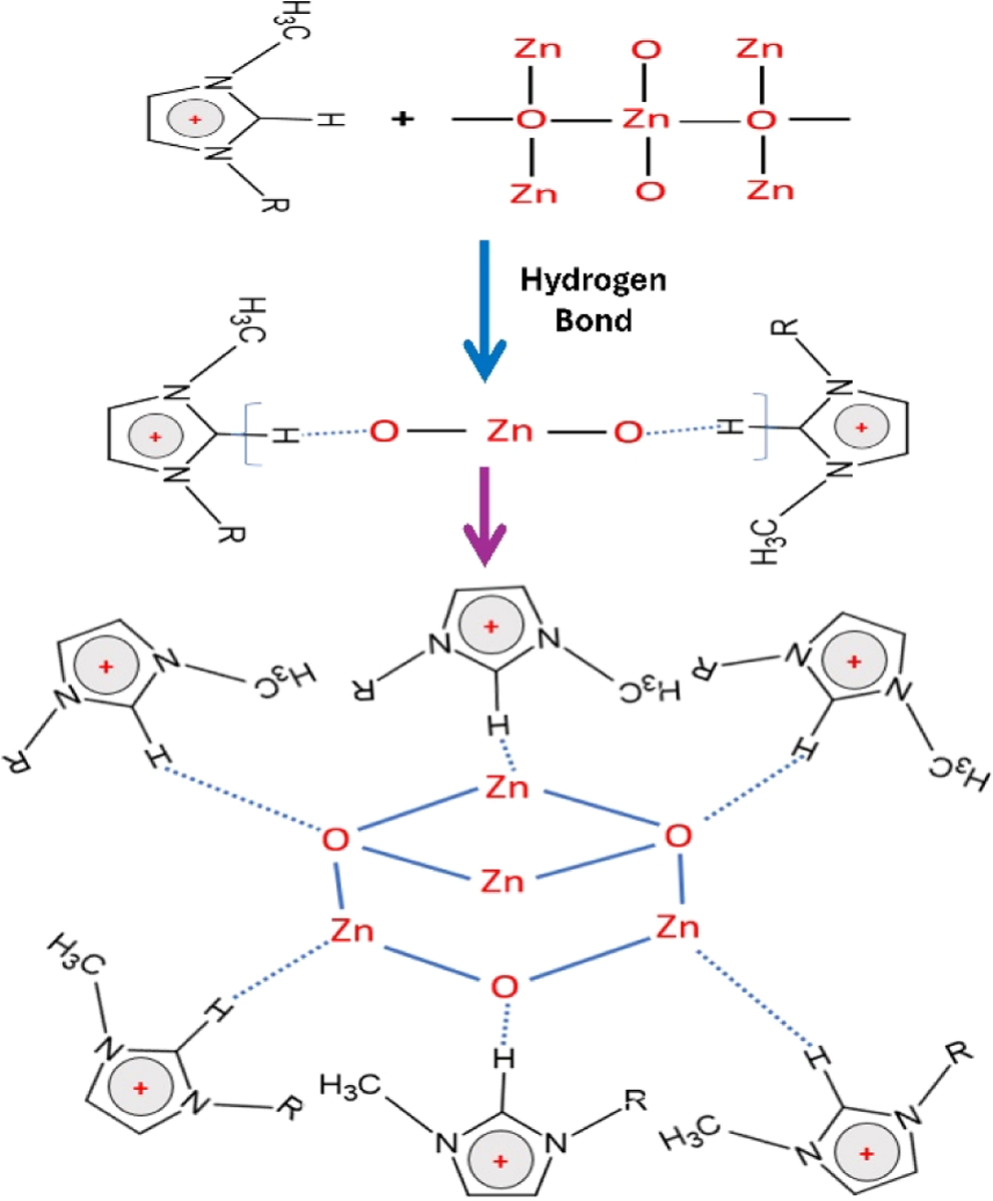
Schematic illustration of ZnO formation with ionic liquid capping.

Figure 12 illustrates the proposed mechanism for ZnO formation in the presence of ionic liquids (ILs). The unique characteristics of ILs, particularly their large cations and anions, enable them to function as self-assembling templates. Acting like surfactants, these ions reduce particle aggregation and facilitate the controlled growth of ZnO nanocrystals. The self-organizing nature of ILs fosters the development of well-defined nanostructures through a “hydrogen bonding-*co*-π stacking” mechanism. In this mechanism, the imidazolium cation of the ILs interacts with the ZnO precursor through hydrogen bonding or electrostatic forces, driving anisotropic crystal growth. Specifically, hydrophilic ionic liquids with longer alkyl chains restrict particle growth due to steric hindrance, resulting in smaller, capsule-shaped ZnO nanostructures.

### 3.4 Mechanism of Methyl Orange Photodegradation

**Figure 13:**
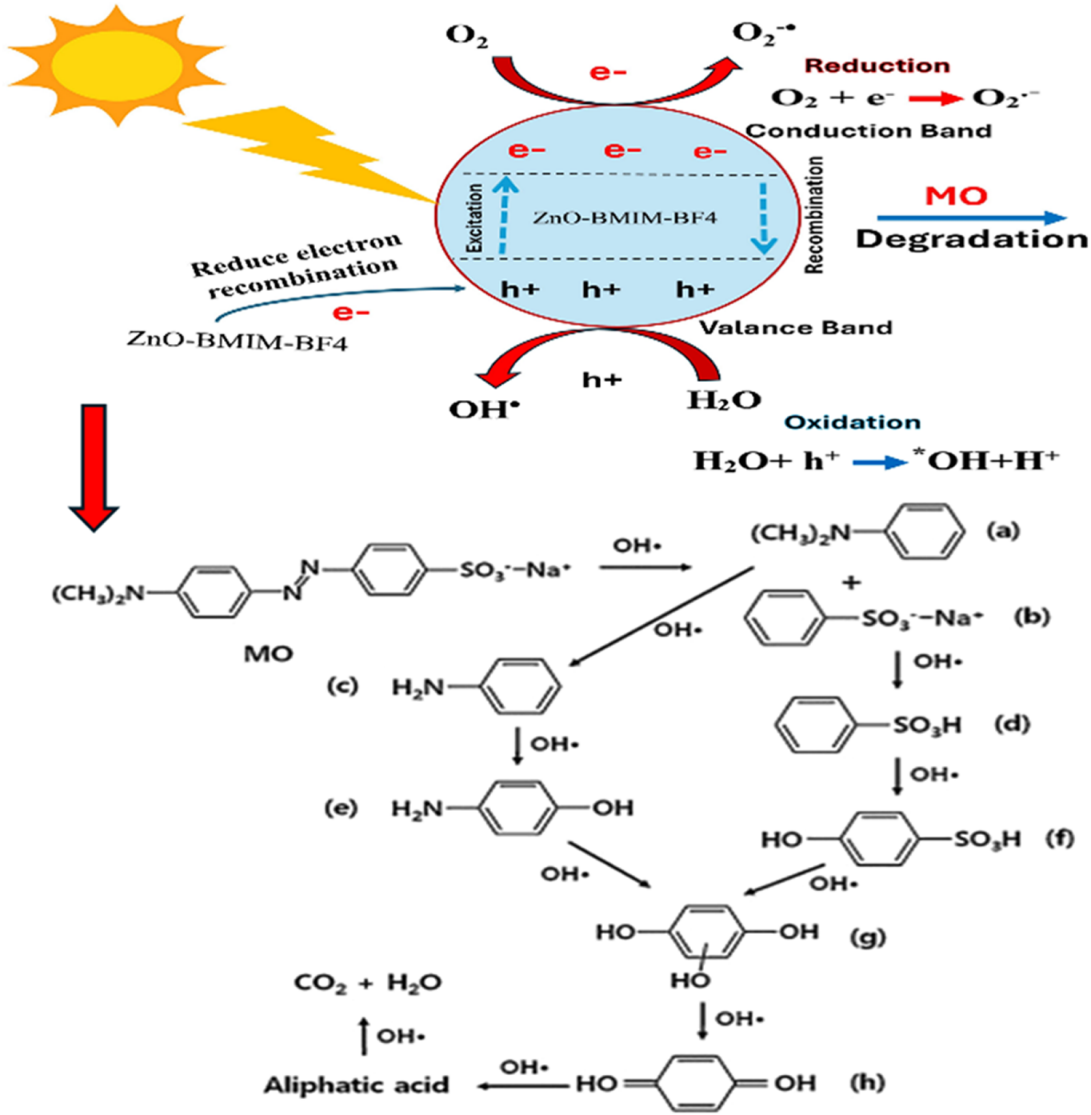
Proposed pathway of the decolorization reaction for methyl orange degradation using the ZnO-BMIM-BF4 photocatalyst.

Figure 13 outlines the decolorization pathway of methyl orange (MO) when treated with the ZnO-BMIM-BF4 system under UV–vis irradiation. The chromophore of MO, containing an azo group, is crucial in this reaction. In the presence of the ZnO-BMIM-BF4 photocatalytic system, reactive species such as ^•^O_2_^–^, ^•^OH and electron–hole pairs (e^–^, h+) are generated, leading to the cleavage of electron-rich sites, particularly the azo nitrogen atom. This process forms intermediate compounds, including dimethylaniline (a) and sodium benzenesulfonate (b). Through subsequent reactions, the dimethyl group is replaced by protons, producing aniline (c) and benzenesulfonic acid (d). Further transformations yield hydroxy aniline (e) and 4-hydroxybenzenesulfonic acid (f), which are eventually broken down into hydroquinone (g) and p-benzoquinone (h). These intermediates are then degraded into smaller molecules such as oxalic acid and carboxylic acid, ultimately decomposing into CO_2_ and H_2_O.^80,81^

In photocatalytic processes, the C–H bonds in ionic liquids (ILs) can act as effective hole scavengers, enhancing the efficiency of the reactions. When a semiconductor absorbs light, it generates electron–hole pairs. The hydrogen atoms in the C–H bonds donate electrons to the photogenerated holes, reducing their availability for recombination with electrons. This reduction in recombination rates allows for more free electrons to participate in the photocatalytic reactions, thereby increasing overall activity. As a result, ILs with active C–H bonds can significantly improve photocatalytic performance by preventing energy loss and boosting reaction efficiency.^82,83^

**Figure 14:**
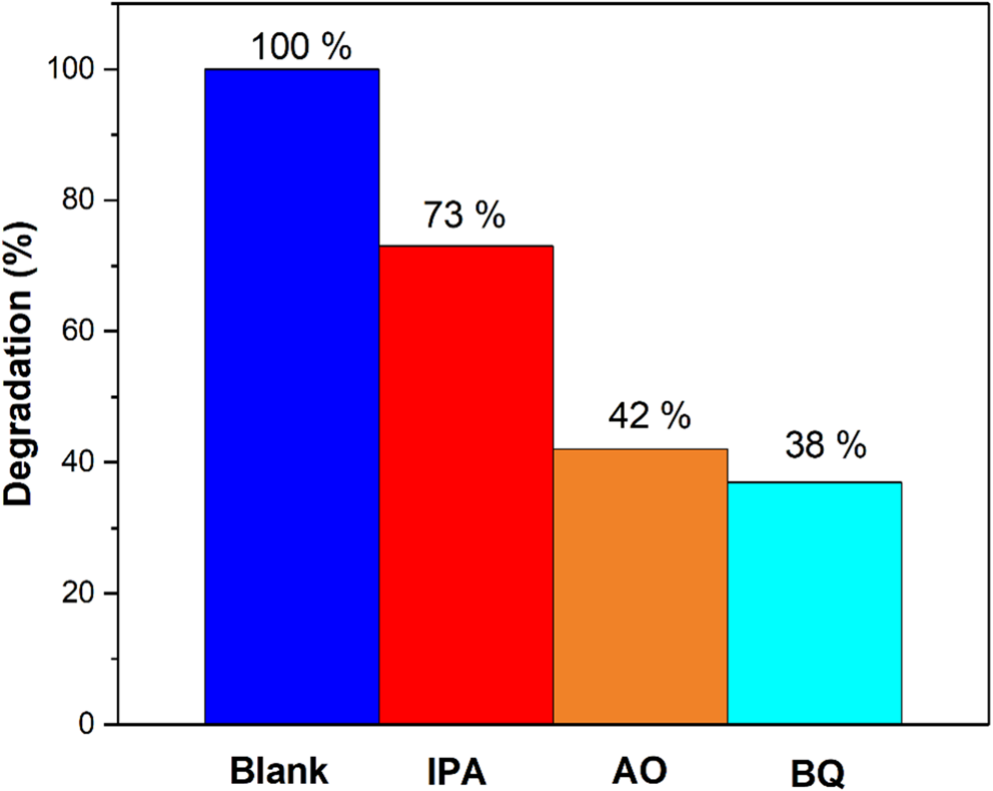
Radical scavenging activity in the decomposition of MO using ZnO-BMIM-BF4 under UV–B irradiation.

To investigate the active free radicals generated in the photocatalytic system, isopropanol (IPA), ammonium oxalate (AO), and benzoquinone (BQ) were introduced as scavengers to capture hydroxyl radicals (^•^OH), holes (h+), and superoxide radicals (^•^O_2_^–^), respectively.^84^ As shown in Figure 14, the addition of these scavengers had varying effects on the photocatalytic efficiency of the ZnO-[BMIM]-BF_4_ sample. The data revealed a significant decrease in the elimination efficiency of MO due to the quenching effect, with BQ notably inhibiting MO removal. The strong response of the ZnO-[BMIM]-BF_4_ sample to BQ and AO indicates that ^•^O_2_^–^ and h+ were the primary active species during the photodegradation process, while ^•^OH served as a secondary, supporting species.

The IL-synthesized ZnO nanocatalysts present several distinct advantages over conventional ZnO nanocatalysts in catalytic processes. One of the key benefits is the improved photocatalytic efficiency. The IL-synthesized ZnO nanoparticles exhibit significantly enhanced photocatalytic activity due to improved charge carrier dynamics, which are influenced by the interaction between the ionic liquids (ILs) and the ZnO’s electronic properties. This results in better overall performance during photocatalytic reactions.

Another notable advantage is controlled synthesis and morphology. During the synthesis process, ILs act as structure-directing agents, enabling the formation of ZnO nanoparticles with well-defined shapes and sizes. This precise control over morphology is crucial for optimizing the catalytic activity of the nanoparticles, making them more effective in various applications.

Furthermore, ILs help in enhancing charge separation within the ZnO nanocatalysts. By reducing electron–hole recombination rates, as indicated by lower photoluminescence (PL) intensities, the presence of ILs increases the availability of charge carriers, which in turn improves the efficiency of photocatalytic reactions.

In terms of stability, IL-synthesized ZnO catalysts, particularly ZnO-BMIM-PF_6_ (1%), show remarkable durability. They maintain high photocatalytic performance over multiple degradation cycles, making them highly suitable for practical applications that require long-term use.

Finally, the IL-assisted synthesis method offers environmental and economic benefits. Ionic liquids have low volatility and can be recycled, making the synthesis process more environmentally friendly. Additionally, the improved efficiency and stability of these catalysts can lead to cost savings in industrial processes, further enhancing their appeal for widespread use.^71,85,86^

## 4 Conclusions

This study investigated the synthesis, characterization, and photocatalytic activity of metal oxide nanoparticles, specifically zinc oxide (ZnO), synthesized in various ionic liquids (ILs). The utilization of ILs, namely [BMIM]-BF_4_, [BMIM]-PF_6_, and [BMIM]-Cl, during synthesis significantly influenced the structural, morphological, and optical properties of the resulting ZnO nanoparticles.

XRD analysis confirmed the successful formation of highly crystalline ZnO nanostructures with a hexagonal wurtzite configuration. The incorporation of ILs was found to induce changes in the crystal lattice, affecting crystallite size and morphology as evidenced by SEM analysis. Notably, the choice of IL played a crucial role in determining particle size and morphology, highlighting the potential for tailoring material properties through IL selection.

Fourier-transform infrared (FT-IR) spectroscopy revealed significant shifts in peak intensities and positions, indicating the active participation of the ionic liquids in the metal oxide network formation.

Optical characterization revealed that IL modification resulted in a red shift in the absorption edge and a reduction in the band gap energy of ZnO. This effect was particularly pronounced for ZnO-[BMIM]-BF_4_, suggesting enhanced light absorption capabilities compared to pure ZnO. The observed changes in optical properties are attributed to the interaction between the IL and ZnO, influencing electronic transitions and band structures.

The photocatalytic performance of the synthesized materials was evaluated through the degradation of MO under UV–B and sunlight irradiation. Remarkably, all IL-modified ZnO catalysts exhibited significantly enhanced photocatalytic activity compared to unmodified ZnO. ZnO-[BMIM]-BF_4_, particularly at concentrations of 1 and 0.5%, demonstrated the highest degradation efficiency, achieving complete removal of Methyl Orange within a short irradiation time.

This enhanced performance is attributed to several factors, including increased surface area, reduced band gap energy, and suppressed electron–hole recombination rates, as evidenced by PL spectroscopy. The ability of ILs to act as a medium for efficient charge transfer and separation further contributes to the improved photocatalytic activity.

Furthermore, the ZnO-BMIM-PF6 (1%) photocatalyst exhibited exceptional stability, maintaining its high degradation efficiency over multiple cycles of use. This stability highlights the potential of these materials for long-term applications in environmental remediation.

In conclusion, this study demonstrates the significant potential of utilizing ILs as a versatile tool for tailoring the properties of metal oxide nanoparticles for enhanced photocatalytic applications. The ability to control particle size, morphology, optical properties, and ultimately photocatalytic performance through IL selection opens up new avenues for designing highly efficient materials for environmental remediation, particularly in the context of dye degradation and wastewater treatment. Further research exploring the use of different ILs, and metal oxide combinations is warranted to fully realize the potential of this promising approach.

## Supporting Information Available

Chemicals and materials; characterization techniques, including Scanning Electron Microscopy (SEM), Fourier Transform Infrared Spectroscopy (FT-IR), UV–Vis spectroscopy, Tauc plot analysis, X-ray Diffraction (XRD) patterns, and Photoluminescence (PL) spectra. Photodegradation of methyl orange (MO) under UV–B and sunlight irradiation; stability tests for photocatalytic degradation; schematic illustration of ZnO formation with ionic liquid capping; proposed pathway for the decolorization reaction of methyl orange degradation; and radical scavenging activity in the decomposition of MO (PDF)
