# Supplementary material for: Metal Oxide Nanoparticles Synthesized in Ionic Liquids: Characterization and Photodegradation of Methyl Orange
Source: ACS Omega. 2025 Mar 6;10(10):9962–75. doi: 10.1021/acsomega.4c07627 (PMC11923637; doi:10.1021/acsomega.4c07627)
Supplement: Supplementary file 1 — ao4c07627_si_001.pdf [file ao4c07627_si_001.pdf]

Metal Oxide Nanoparticles Synthesized in Ionic Liquids: Characterization and Photodegradation of Methyl Orange

*Mohamedalameen H. A. Hussain, Gulin Selda Pozan SOYLU**

Istanbul University-Cerrahpaşa, Faculty of Engineering, Chemical Engineering Department, Avcilar, 34320 Istanbul, Turkey.

* Correspondence: gpozan@iuc.edu.tr

Text S1. Chemicals and materials

Text S2. Characterizations

Figure S1. XRD of synthesized: (a) Pure ZnO nanoparticles and ZnO nanoparticles in (b) [BMIM]BF_4 (%1), (c) [BMIM]Cl (1%), and (d) [BMIM]PF_6 (1%).

Figure S2. Analysis of the (101) peak shifts of (a) Pure ZnO nanoparticles and ZnO nanoparticles in (b) [BMIM]BF_4 (%1), (c) [BMIM]Cl (1%), and (d) [BMIM]PF_6 (1%).

Figure S3. XRD of synthesized: (a) Pure ZnO nanoparticles and ZnO nanoparticles in (b) [BMIm]BF_4 (0.5%), (c) [BMIM]BF_4 (1%), and (d) BMIm]BF_4 (2%).

Figure S4. Analysis of the (101) peak shifts of (a) Pure ZnO nanoparticles and ZnO nanoparticles in (b) [BMIm]BF_4 (0.5%), (c) [BMIM]BF_4 (1%), and (d) BMIm]BF_4 (2%).

Figure S5. SEM images of synthesized: (a) Pure ZnO nanoparticles and ZnO nanoparticles in (b) [BMIm]BF_4 (1%), (c) [BMIm]Cl (1%), (d) [BMIm] PF_6 (1%), (e) [BMIM]-BF_4 (0.5%), (f) [BMIM]-BF_4 (2%).

Figure S6.

(A) FT-IR spectra of synthesized ZnO nanoparticles: (a) pure ZnO, (b) ZnO in [BMIM]-BF_4 (1%), (c) ZnO in [BMIM]-Cl (1%), and (d) ZnO in [BMIM]-PF_6 (1%).

(B) FT-IR spectra of synthesized ZnO nanoparticles at varying [BMIM]-BF_4 concentrations: (a) pure ZnO, (b) ZnO in [BMIM]-BF_4 (0.5%), (c) ZnO in [BMIM]-BF_4 (1%), and (d) ZnO in [BMIM]-BF_4 (2%).

Figure S7(a) and (b). UV–Vis absorption spectra of pure ZnO nanoparticles and ZnO photocatalysts synthesized in various ionic liquids.

Figure S8. Tauc plots of pure ZnO nanoparticles and ZnO photocatalysts synthesized in different ionic liquids.

Figure S9. PL spectra of pure ZnO nanoparticles and ZnO photocatalysts synthesized in different ionic liquids.

Figure S10.

(a) Photodegradation of methyl orange (MO) under UV-B irradiation by pure ZnO nanoparticles and ZnO photocatalysts synthesized in different ionic liquids.

(b) Photodegradation of methyl orange (MO) under sunlight irradiation by pure ZnO nanoparticles and ZnO photocatalysts synthesized in different ionic liquids.

Figure S11. Stability tests for the photocatalytic degradation under sunlight and UV-B irradiation of MO on ZnO-BMIM-PF6(1%) photocatalyst.

Figure S12. Schematic illustration of ZnO formation with ionic liquid capping.

Figure S13. Proposed pathway of the decolorization reaction for methyl orange degradation using the ZnO-BMIM-BF4 photocatalyst.

Figure S14. Radical scavenging activity in the decomposition of MO using ZnO-BMIM-BF4 under UV-B irradiation.

Text S1. Chemicals and materials

1-Butyl-3-methylimidazolium tetrafluoroborate, 1-Butyl-3-Methylimidazolium Hexafluorophosphate, 1-Butyl-3-Methylimidazolium Iodide, Nitrate Hexahydrate ($Zn(NO_3)_2{\cdot}6H_2O$), Sodium Hydroxide (NaOH), Methanol Absolute, Hydrochloric Acid (HCl), Ethanol, ultrapure water.

Text S2. Characterizations

The specific surface areas of the samples were determined using nitrogen adsorption-desorption isotherm measurements at 77 K (Quantachrome Instrument). Prior to the actual measurements, the samples were degassed at 200 ˚C for 2 hours.

X-ray powder diffraction analysis was performed on the specimens utilizing a Rigaku D/Max-2200 diffractometer, employing Cu Kα radiation with a wavelength (λ) of 1.540 Å. The scanning range for the samples encompassed 10 to 80 degrees 2θ, at a scanning rate of 2 degrees per minute. To determine the sizes of the crystalline domains, the Scherrer equation was applied:

$$t = \frac{c\lambda}{(B * cos\theta)} \qquad Eq.\ (1)$$

where λ represents the X-ray wavelength in Ångstroms (Å), B corresponds to the full width at half maximum (FWHM), θ represents the Bragg angle, C is a shape-dependent factor (assumed to be unity), and t denotes the crystallite size in Ångstroms (Å).

Fourier transform infrared (FTIR) spectra were acquired under ambient conditions using a Perkin Elmer Precisely Spectrum One spectrometer with KBr as the diluent. The measurements were conducted at a resolution of 4 cm^{-1}, and each spectrum was obtained by averaging 100 scans.

The light absorption properties of the photocatalysts were investigated through UV-Vis Ocean optics model DH-2000-BAL spectrophotometer. The spectra results were recorded in the 280–1000 nm range using halogen and deuterium lamps as light sources. and the absorption band gap energy (Eg) was determined utilizing the Kubelka-Munk function. SEM images of the photocatalyst samples were acquired at various magnifications using a Zeiss EVO MA10 SEM instrument (Germany), operating at an acceleration voltage of 10 kV.

Photoluminescence (PL) spectra were acquired at room temperature using a Perkin-Elmer LS-50 fluorescence spectrophotometer. The sample was first dispersed in ethanol via ultrasonication. A Xenon lamp served as the excitation source, and an excitation wavelength of 325 nm was used for all measurements.

The photocatalytic performance of the catalysts was assessed through the degradation of methyl orange. To conduct these experiments, we utilized the LUZCHEM LZC-5 photoreactor system equipped with UV lamps, specifically 64 W UV-B, 64 W UV-A, and 64 W and 100 W halogen lamps as light sources. In a typical experimental procedure, 100 mg of the catalyst was dispersed in 50 mL of aqueous dye solutions with an initial concentration of 25 mg/L and maintained at a neutral pH. This mixture was subjected to magnetic stirring after undergoing 10 min. of

ultrasonication to ensure the establishment of an adsorption-desorption equilibrium between the catalyst and the solution. This step was performed in the dark. Subsequently, the aqueous dye solution was exposed to different time intervals of illumination over a span of 2 hours. After the photocatalytic treatment, the solution was filtered using a membrane filter with a pore size of 0.45 μm to separate the catalyst from the dye solution. The filtered solution was then further analyzed using UV-Vis absorption spectroscopy.

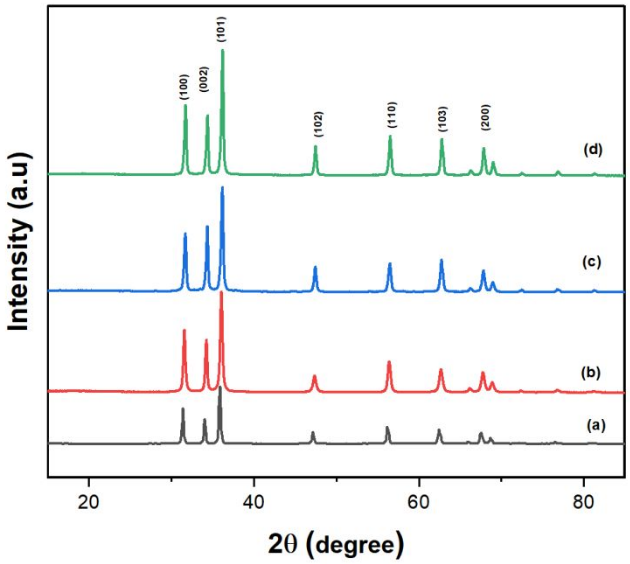

Figure S1. XRD of synthesized: (a) Pure ZnO nanoparticles and ZnO nanoparticles in (b) [BMIM]BF_4 (%1), (c) [BMIM]Cl (1%), and (d) [BMIM]PF_6 (1%).

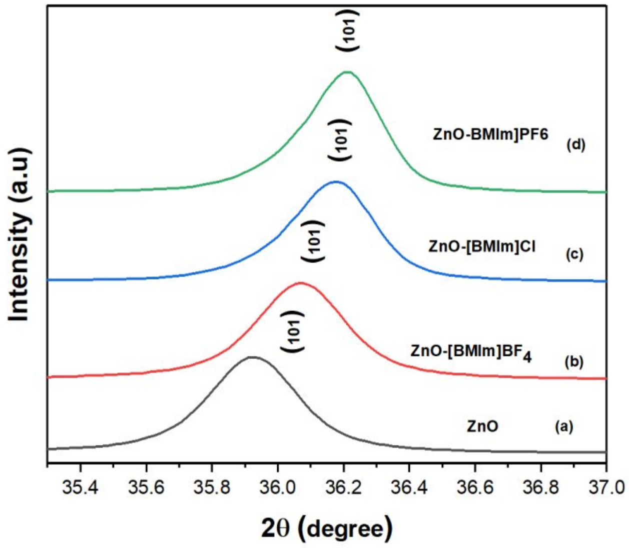

Figure S2. Analysis of the (101) peak shifts of (a) Pure ZnO nanoparticles and ZnO nanoparticles in (b) [BMIM]BF_4 (%1), (c) [BMIM]Cl (1%), and (d) [BMIM]PF_6 (1%).

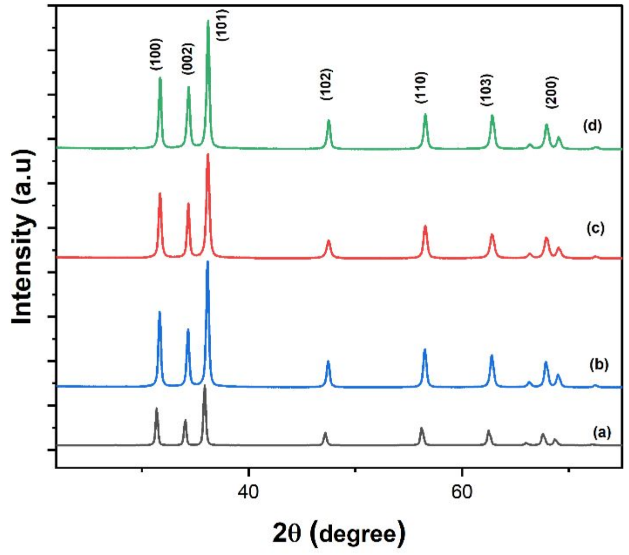

Figure S3. XRD of synthesized: (a) Pure ZnO nanoparticles and ZnO nanoparticles in (b) [BMIm]BF_4 (0.5%), (c) [BMIM]BF_4 (1%), and (d) BMIm]BF_4 (2%).

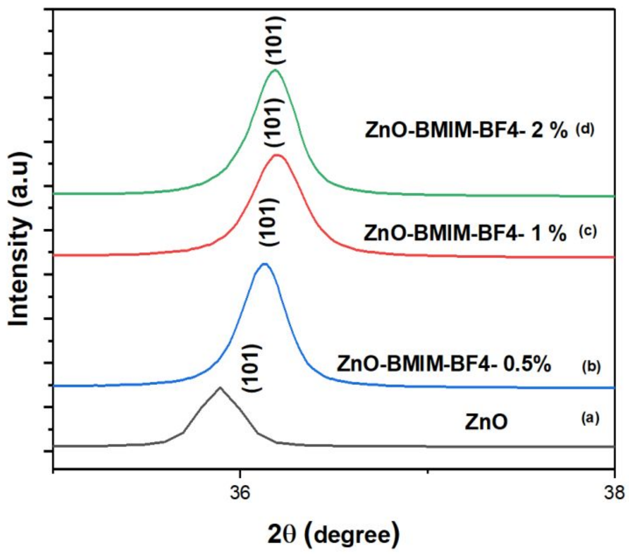

Figure S4. Analysis of the (101) peak shifts of (a) Pure ZnO nanoparticles and ZnO nanoparticles in (b) [BMIm]BF_4 (0.5%), (c) [BMIM]BF_4 (1%), and (d) BMIm]BF_4 (2%).

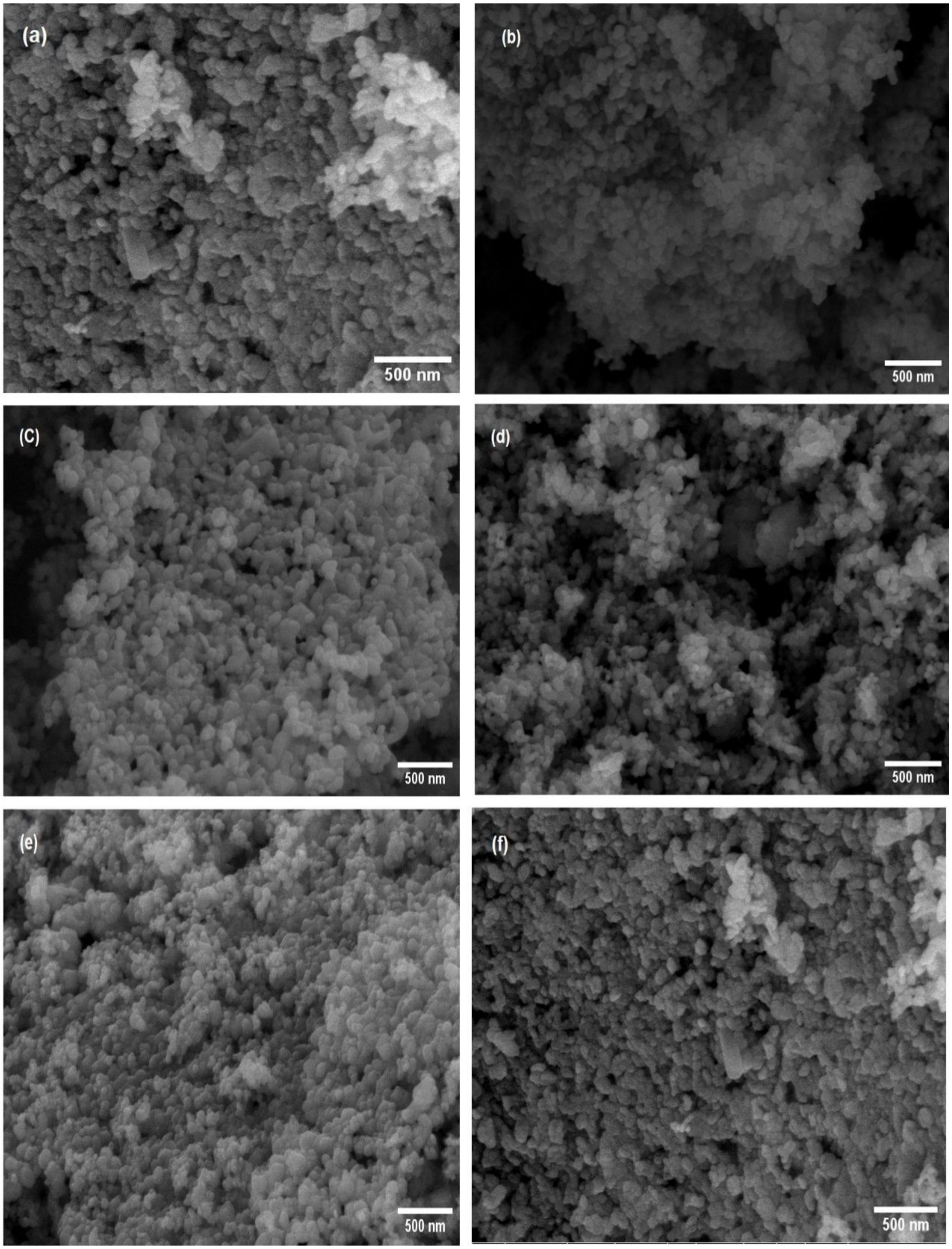

Figure S5. SEM images of synthesized: (a) Pure ZnO nanoparticles and ZnO nanoparticles in (b) [BMIm]BF_4 (1%), (c) [BMIm]Cl (1%), (d) [BMIm] PF_6 (1%), (e) [BMIM]-BF_4 (0.5%), (f) [BMIM]-BF_4 (2%). Magnification is 100000 X for a scale bar of 500 nm.

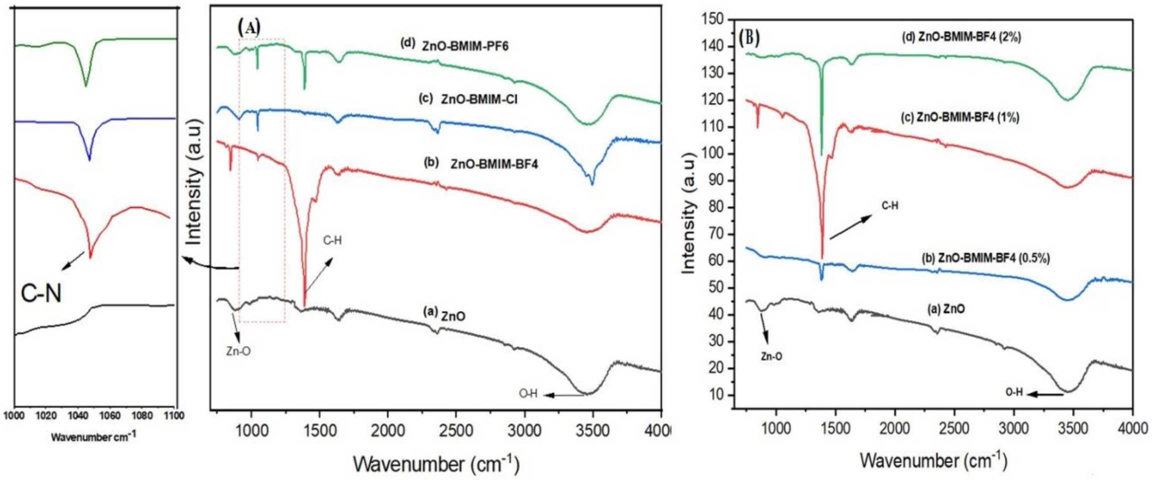

Figure S6.

(A) FT-IR spectra of synthesized ZnO nanoparticles: (a) pure ZnO, (b) ZnO in [BMIM]-BF_4 (1%), (c) ZnO in [BMIM]-Cl (1%), and (d) ZnO in [BMIM]-PF_6 (1%).

(B) FT-IR spectra of synthesized ZnO nanoparticles at varying [BMIM]-BF_4 concentrations: (a) pure ZnO, (b) ZnO in [BMIM]-BF_4 (0.5%), (c) ZnO in [BMIM]-BF_4 (1%), and (d) ZnO in [BMIM]-BF_4 (2%).

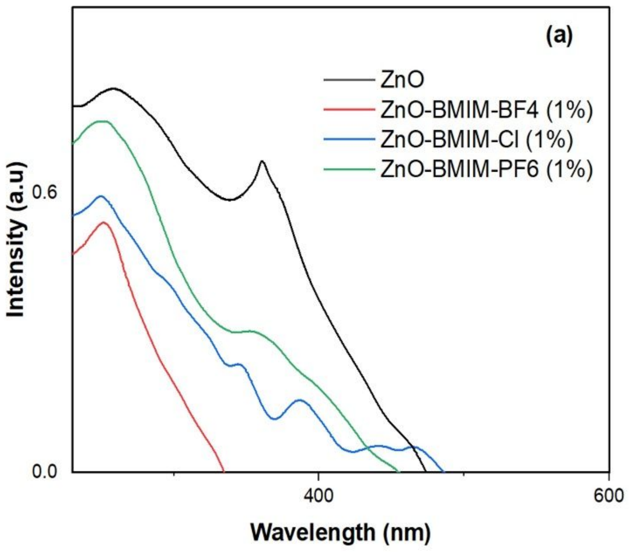

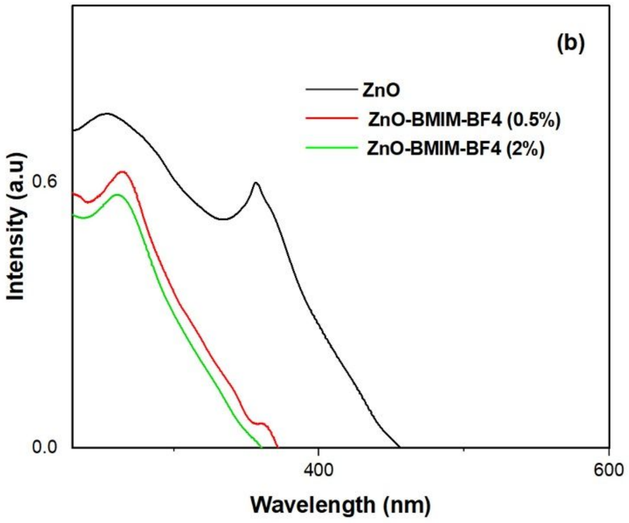

Figure S7(a) and (b). UV–Vis absorption spectra of pure ZnO nanoparticles and ZnO photocatalysts synthesized in various ionic liquids.

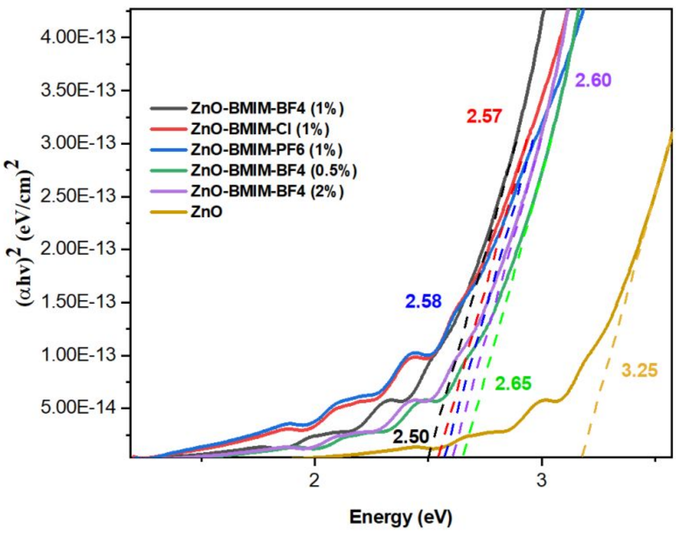

Figure S8. Tauc plots of pure ZnO nanoparticles and ZnO photocatalysts synthesized in different ionic liquids.

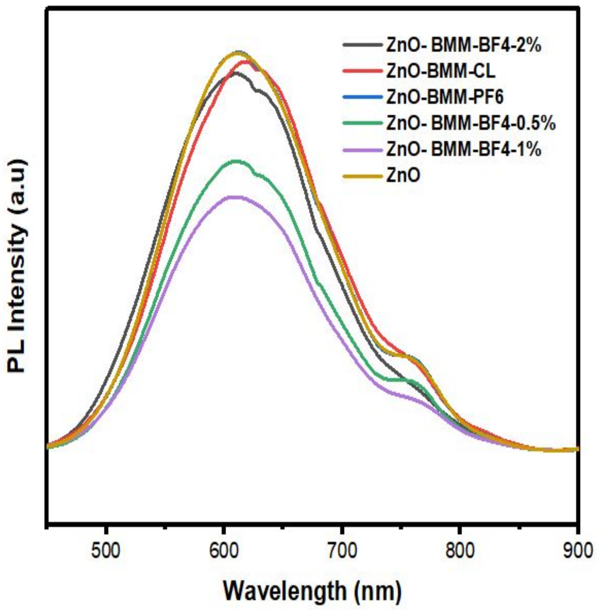

Figure S9. PL spectra of pure ZnO nanoparticles and ZnO photocatalysts synthesized in different ionic liquids.

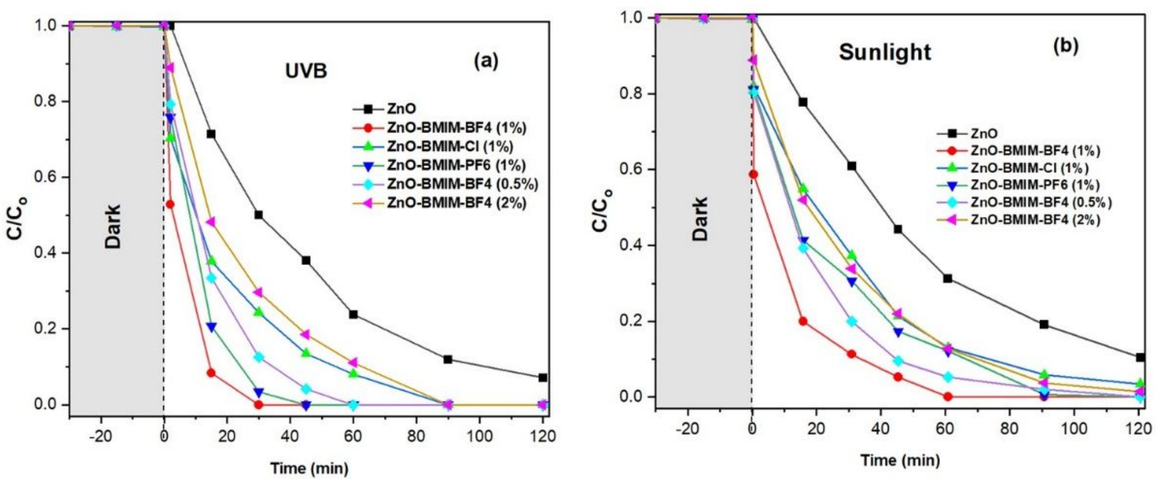

Figure 10.

(a) Photodegradation of methyl orange (MO) under UV-B irradiation by pure ZnO nanoparticles and ZnO photocatalysts synthesized in different ionic liquids.

(b) Photodegradation of methyl orange (MO) under sunlight irradiation by pure ZnO nanoparticles and ZnO photocatalysts synthesized in different ionic liquids.

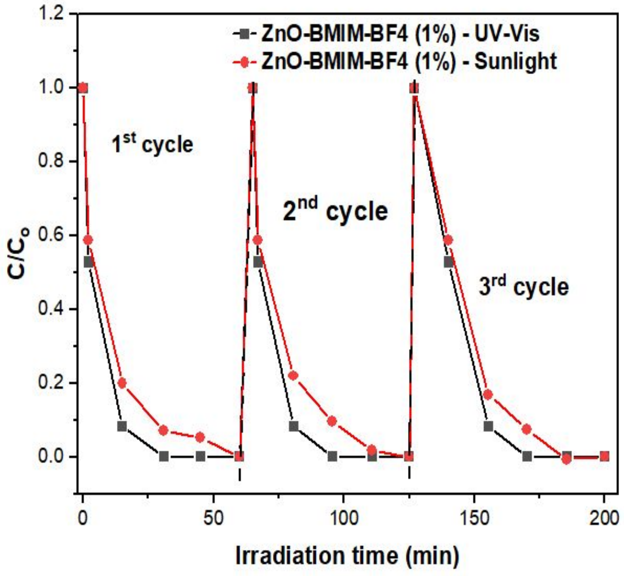

Figure S11. Stability tests for the photocatalytic degradation under sunlight and UV-B irradiation of MO on ZnO-BMIM-PF6(1%) photocatalyst.

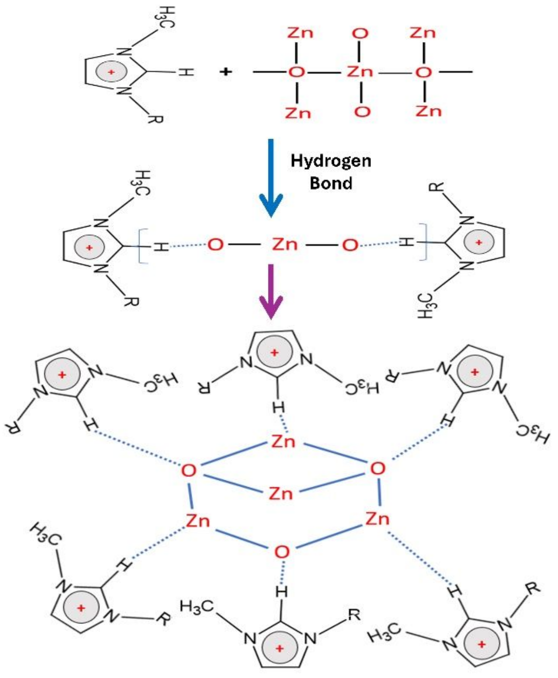

Figure S12. Schematic illustration of ZnO formation with ionic liquid capping.

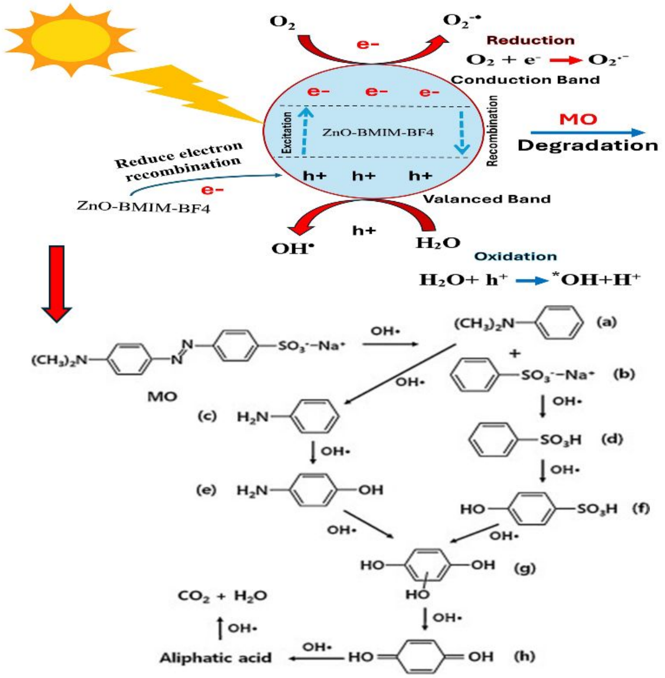

Figure S13. Proposed pathway of the decolorization reaction for methyl orange degradation using the ZnO-BMIM-BF4 photocatalyst.

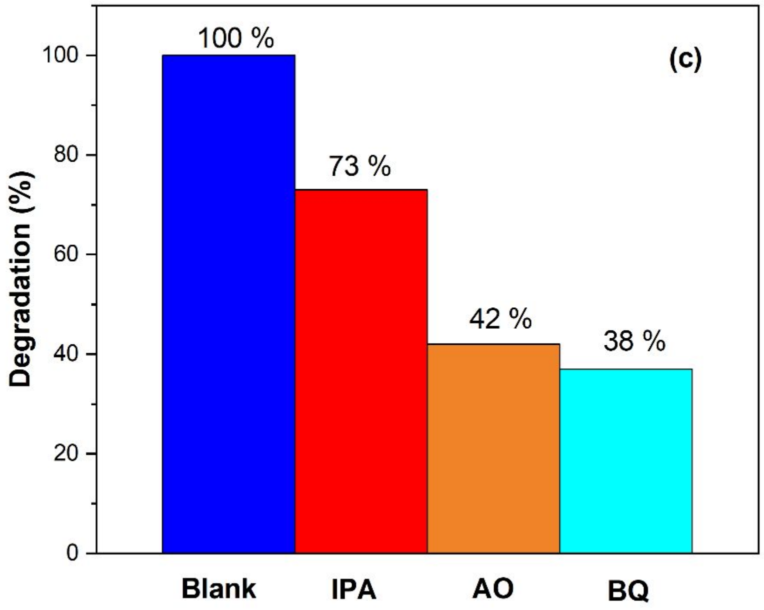

Figure S14. Radical scavenging activity in the decomposition of MO using ZnO-BMIM-BF4 under UV-B irradiation.
